# Tgfb3 and Mmp13 regulated the initiation of liver fibrosis progression as dynamic network biomarkers

**DOI:** 10.1111/jcmm.16140

**Published:** 2020-12-02

**Authors:** Jinsheng Guo, Weixin Liu, Zhiping Zeng, Jie Lin, Xingxin Zhang, Luonan Chen

**Affiliations:** ^1^ Department of Gastroenterology and Hepatology Zhong Shan Hospital Fu Dan University Shanghai Institute of Liver Diseases Shanghai China; ^2^ Key Laboratory of Systems Biology Shanghai Institute of Biochemistry and Cell Biology Center for Excellence in Molecular Cell Science Chinese Academy of Sciences Shanghai China; ^3^ Key Laboratory of Systems Biology Hangzhou Institute for Advanced Study University of Chinese Academy of Sciences Chinese Academy of Sciences Hangzhou China; ^4^ School of Life Science and Technology ShanghaiTech University Shanghai China

**Keywords:** dynamic network biomarkers, liver fibrosis, tipping point, transition

## Abstract

Liver fibrogenesis is a complex scar‐forming process in the liver. We suggested that the liver first responded to chronic injuries with gradual changes, then reached the critical state and ultimately resulted in cirrhosis rapidly. This study aimed to identify the tipping point and key molecules driving liver fibrosis progression. Mice model of liver fibrosis was induced by thioacetamide (TAA), and liver tissues were collected at different time‐points post‐TAA administration. By dynamic network biomarker (DNB) analysis on the time series of liver transcriptomes, the week 9 post‐TAA treatment (pathologically relevant to bridging fibrosis) was identified as the tipping point just before the significant fibrosis transition, with 153 DNB genes as key driving factors. The DNB genes were functionally enriched in fibrosis‐associated pathways, in particular, in the top‐ranked DNB genes, Tgfb3 negatively regulated Mmp13 in the interaction path and they formed a bistable switching system from a dynamical perspective. In the *in vitro* study, Tgfb3 promoted fibrogenic genes and down‐regulate Mmp13 gene transcription in an immortalized mouse HSC line JS1 and a human HSC line LX‐2. The presence of a tipping point during liver fibrogenesis driven by DNB genes marks not only the initiation of significant fibrogenesis but also the repression of the scar resolution.

**Abbreviations**
CI: composite index
CTGF: connective tissue growth factor
DEGs: differentially expressed genes
DNB: dynamic network biomarker
ECM: extracellular matrix
HCA: hierarchical analysis
HISAT: hierarchical indexing for spliced alignment of transcripts
HSC: hepatic stellate cells
IPA: ingenuity pathway analysis
Itgb2L: integrin subunit beta 2
KEGG: Kyoto encyclopaedia of genes and genomes
Mmp13: matrix metalloproteinase 13
NAFLD: non‐alcoholic fatty liver disease
PCA: principle component analysis
PCCs: Pearson's correlation coefficients
TAA: thioacetamide
Tgfb1: transforming growth factor β1
Tgfb3: transforming growth factor β3
TIMPs: tissue inhibitors of metalloproteinases
α‐SMA: α‐smooth muscle actin

## 1 INTRODUCTION

Liver fibrosis is a common scar‐forming process in the liver in response to various chronic insults. It is not an independent disease but a conceptually important stage of liver cirrhosis, during which the fibrotic scar is generally considered more soluble, and normal liver function and structure are likely preserved after effective treatment. The fibrogenic process is often triggered and accompanied by cell necrosis and inflammatory infiltration, which subsequently activate inflammatory and fibrogenic signalling cascades.^1^, ^2^ With injury and fibrosis progression, the normal anatomical lobules of the liver are gradually replaced by architecturally abnormal nodules separated by fibrous tissue, leading to cirrhosis and eventually functionally decompensated cirrhosis with fatal complications.^3^ This occurs when the treatment is limited and overall prognosis is poor. Hepatic fibrosis itself and cirrhosis in compensated phase not only are lack of accurate non‐invasive serum biomarkers but also have no distinct symptoms. The Metavir staging systems have been used for histological assessment of chronic liver diseases on the base of liver biopsy. Significant fibrosis refers to a METAVIR score of F2 or greater, whereas METAVIR F4 denotes cirrhosis. This is likely to have important implications especially in the early phases of the fibrogenic process when the fibrosis regression is higher because of the absence of significant changes in the tissue architecture.^4^ While there is consistent evidence of the reversibility of fibrosis in non‐cirrhotic liver, the determinants of fibrosis progression as well as regression in cirrhosis are not clear, and the so‐called ‘tipping point’ in cirrhosis is not clearly established. Thus, it is crucial to identify when and why fibrosis becomes irreversible to effectively prevent the development of advanced cirrhosis.

Many *in vivo* and *in vitro* studies have revealed that hepatic fibrogenesis is a highly dynamic process attributable to the interactions between hepatic parenchymal and non‐parenchymal cells as well as the complex networks within cytokines and other intracellular/extracellular signals. Various signalling pathways and downstream transcriptional factors were involved in inflammatory and hepatic fibrogenesis. Possible core pathway components include αν integrin, transforming growth factor β (Tgfb) and its downstream signalling effector molecule, for example connective tissue growth factor (CTGF). The activation of hepatic stellate cells (HSC) is the central event of hepatic fibrogenesis, driven by the stimulation of cell death (apoptosis and necrosis), inflammation and oxidative stress, extracellular matrix (ECM) damage and reconstruction, inflammation and fibrogenic cytokines. Upon activation, cells transform to a myofibroblast phenotype with high proliferation activity, express α‐smooth muscle actin (α‐SMA) and ECM components; obtain cell contractility, chemotaxis and migration properties; and produce a large amount of growth factors and profibrogenic cytokines to promote fibrosis (eg Tgfb and platelet‐derived growth factor).^5^

Dynamic analysis based on high‐throughput data provides new concepts and methods for identifying early warning signals of complex diseases, including liver fibrosis/cirrhosis and revealing basic mechanisms of disease initiation and progression at the network level. A novel model‐free method based on nonlinear dynamic theory, termed dynamic network biomarker (DNB), has been developed to characterize the tipping point just before the critical transition during the progression of complex diseases.^6^, ^7^ Based on DNB theory, for many chronic diseases, there existed a sudden shift during the process of gradual health deterioration that results in a drastic transition to a disease state. In other words, the progression of a disease is divided into three phases, that is normal, pre‐disease (tipping point or critical state) and disease states.^8^, ^9^, ^10^, ^11^, ^12^, ^13^, ^14^ Unlike the normal and disease states, in which the complex systems are usually robust to perturbations in such stable states, a pre‐disease state has low resilience and robustness. After crossing pre‐disease state, it becomes difficult to return to the normal state because of state stability. Thus, to prevent disease deterioration, identifying such pre‐disease state or tipping point is of great importance on both preventive and predictive medicine. This can be further exploited to detect the driving molecules or DNB genes during disease progression. DNB genes are a group of molecules with strong collective fluctuations, appearing only at the ‘tipping point’ of a homeostatic system, thereby considering as the key factors to drive the critical transition. In particular, the standard deviations (SD) and Pearson's correlation coefficients (PCCs) of DNB genes are both drastically increased at the tipping point just before the transition during the disease progression. A quantitative index, termed composite index (CI), for the DNB genes can be derived for quantifying the tipping point as well as its driving molecules.^9^, ^10^ The DNB method has been successfully applied to real‐world biological data to identify the early warning signals of the sudden deterioration of several complex diseases such as cancer and metabolic disease.^15^, ^16^, ^17^

Progression of liver fibrosis is generally a complex process, triggered by many related genes. Dysfunction of these genes is involved in a wide range of biological mechanisms, which can be used for characterizing the tipping point of critical transition from liver damage to liver fibrotic stage. This study aims to discover the tipping point with its key genes driving liver fibrosis progression by DNB analysis on liver transcriptomes. In addition, we further analyse the switching mechanism of the liver fibrosis by constructing its bistable model of key genes during the dynamic process. Clearly, the results will help not only to reveal the molecular mechanism of liver fibrosis/cirrhosis but also to identify new potential therapeutic targets and molecular markers for non‐invasive diagnosis of liver fibrosis.

## 2 MATERIALS AND METHODS

### 2.1 Animal models of hepatic fibrosis and preparation of time‐series liver sample

The study protocol was approved by the committee on the use of live animals for teaching and research of Zhong Shan Hospital (No. 2011‐131), Fu Dan University, and was performed in accordance with the National Institutes of Health (NIH) guide for the care and use of Laboratory animals (NIH publication 86‐23 revised 1985).

Male C57BL/6J mice approximately 6‐week‐old and weighed 20‐25 g were obtained from the Animal Research Center of Fu Dan University (Shanghai, China). They were fed a standard laboratory diet and housed in standard micro‐isolator cages inside a room with well‐regulated temperature (22 ± 1°C), humidity (65%‐70%) and day/night cycle (12 hours/12 hours). An experimental model of hepatic fibrosis was employed by intraperitoneal injections of thioacetamide (TAA, 100 mg/kg, three times per week) in mice for 17 weeks. Control mice received the same volume of normal saline intraperitoneally. Liver samples of each mouse were collected from 3‐5 mice in the treatment and control groups at different time‐points post‐TAA treatment and stored at −80±°C for RNA and protein analysis. A part of each liver sample was fixed in 10% buffered formalin for histological assessment of the progression of liver fibrosis.

### 2.2 RNA sequencing of time‐series sample and extraction of gene expression data

**FIGURE 1:**
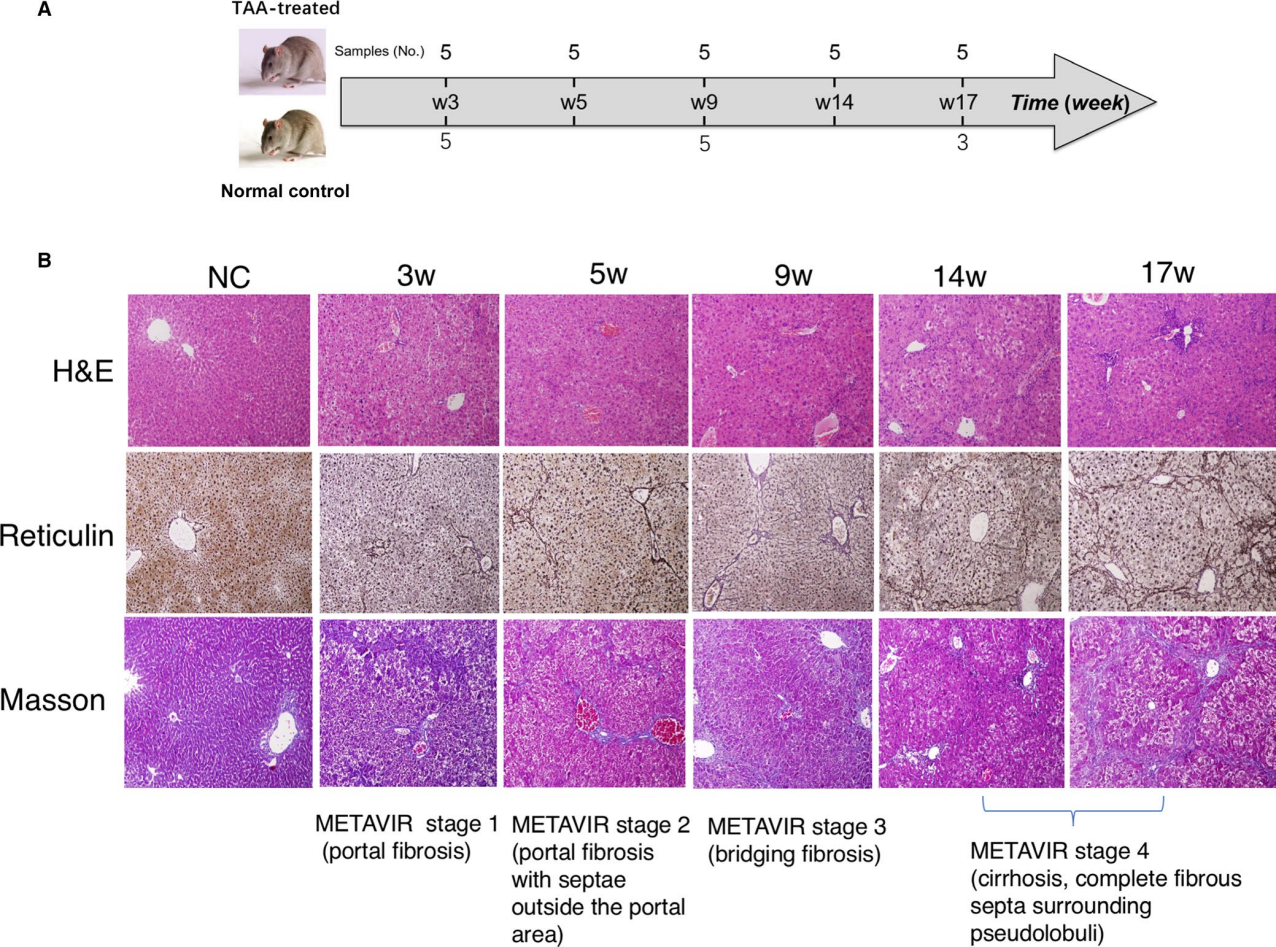
Establishment of mice model of liver fibrosis and assessing fibrosis progression. **(A)** Induction of experimental liver fibrosis by thioacetamide (TAA) in mice. Liver sections were prepared from liver tissue samples of normal mice (NC) and TAA‐treated mice for 3, 5, 9, 14 and 17 weeks (3w, 5w, 9w, 14w, 17w, respectively, N = 5 per group). (B) Morphological examination for the progression of liver fibrosis by haematoxylin and eosin (H&E), Reticulin (Reticulin) and Masson's trichrome (Masson) staining. The assessment of liver fibrosis stages as judged by histological descriptions of METAVIR staging system were marked below the figures. Liver specimens of mice with TAA treatment for 9 weeks shown bridging fibrosis and were relevant to METAVIR stage 3. Magnification was 10×

To construct the time‐series samples, we sequenced the transcriptome of five TAA‐treated mice at five different time‐points (Figure 1A) by RNA sequencing technology. To reduce the influence of development of mice growth, we also sequenced the transcriptome of three normal control mice at weeks 3, 9 and 17.

The experiments of Library construction and RNA Sequencing were completed at centre of Novelbio laboratory (Shanghai, China). Briefly, total RNA was extracted with TRIzol reagent (Invitrogen, Carlsbad, CA, USA) and purified using RNeasy Mini Kit (Qiagen, Valencia, CA). The complementary DNA (cDNA) libraries for single‐end sequencing were prepared using Ion Total RNA‐Seq Kit v2.0 (Life Technologies) according to the manufacturer's instructions. The cDNA libraries were then processed for the Proton Sequencing process according to Ion PI Sequencing 200 Kit v2.0 (Life Technologies). After quality check for sequence reads, we performed sequence alignment using hierarchical indexing for spliced alignment of transcripts (HISAT) for mapping RNA‐seq reads to the reference (https://www.ncbi.nlm.nih.gov/assembly/GCF_000001635.26).^18^ The raw expressed read counts of each sample at each time‐point were then extracted by HTseq count program, which is a Python library to facilitate rapid development of scripts for processing and analysing high‐throughput sequencing data.^19^ Differentially expressed genes (DEGs) were calculated by DEseq2 for all adjacent time‐points of mice (TAA‐treated mice and normal control mice) and TAA‐treated mice versus normal control mice at the same time‐point, with a threshold of fold change > 2 or < 0.5, and false discovery rate (FDR) < 0.05.^20^

### 2.3 Dynamic network biomarker (DNB) analysis

To detect the tipping point and its leading genes in the TAA‐induced animal model during liver fibrogenesis, we used DNB analysis methodology,^10^, ^11^ which can be implemented in time‐series high‐throughput omics data such as RNA sequencing. Based on the DNB theory, a dominant group of molecules, that is DNB genes, are selected if they satisfied the following three criteria among the observed data: (a) Pearson correlation coefficients (*PCC_i_*) of molecules in this DNB (dominant group) drastically increase; (b) Pearson correlation coefficients (*PCC_o_*) between molecules in the dominant group and those outside the group significantly decrease; and (c) standard deviations (*SD_i_*) for any member of this dominant group significantly increase. To obtain a strong signal in the pre‐disease state while considering the above three criteria, the composite index (*CI*) is calculated:

$$CI=\frac{PCC_{i}\times SD_{i}}{PCC_{o}}$$

where *PCC_i_* is the average *PCC* (absolute value) of the dominant group, *PCC_o_* is the average *PCC* (absolute value) between molecules in the dominant group and outside the group, and *SD_i_* is the average standard deviation of the dominant group. According to the DNB theory, the *CI* is expected to increase sharply once the biological system approaches a critical period or tipping point. Thus, it can serve as an effective early warning signal to identify the pre‐disease state as well as the DNB genes.

### 2.4 Functional analysis

Functional enrichment analysis aims to identify classes of molecules (genes or proteins) that are over‐represented in a set of predefined molecules and to predict its association with disease phenotypes. In this study, we performed this method to uncover potential fibrosis‐associated biological functions affected by the identified molecules by mapping the molecules into known molecule sets with functional annotation. Two databases, KEGG and Ingenuity Pathway Analysis (IPA, a database that contains millions of documented and published molecular interactions; http://www.ingenuity.com) were used for canonical pathway detection.^21^ Enrichment of specific molecules in each biological process or pathway was estimated. Significantly enriched functions were chosen if the corresponding p‐value was below a threshold (0.05). Significant DNBs in DEGs were ranked based on their enrichment in IPA and KEGG pathways associated with liver fibrogenesis.

### 2.5 Histological staining and fibrotic staging

Liver histology and fibrosis stages were observed by haematoxylin and eosin (H&E), Masson's trichrome and reticulin staining. Formalin‐fixed liver tissues were embedded in paraffin. Morphological examination was performed with H&E staining for histological assessment of the progression of liver fibrosis. Masson's trichrome and reticulin staining was used to demonstrate fibrotic changes and collagen deposition.

### 2.6 Cell Culture

An immortalized wild‐type mouse HSC line JS1 and a human HSC line LX‐2 were used to test the profibrogenic function of Tgfb3.^22^, ^23^ The cells were maintained in Dulbecco's Modified Eagle's Medium (Gibco, Invitrogen) supplemented with 10% foetal bovine serum (Gibco, Invitrogen) and antibiotics at 37°C in a 5% CO2 humidified atmosphere. In certain experiments, JS1 and LX2 cells were treated with recombinant active Tgfb1 and Tgfb3 protein (Abcam, Cambridge, MA, USA) at a final concentration of 10 ng/mL, or PBS vehicle for 24 or 48 hours, and then collected for RNA or protein analysis.

### 2.7 Real‐time quantitative polymerase chain reaction (RT‐qPCR)

Total RNA was isolated from liver tissues or cells using TRIzol reagent (Invitrogen, Carlsbad, CA) according to the manufacturer's instructions and reversely transcribed into cDNA using PrimeScript RT Master Mix (Takara, Bio, Shiga, Japan) on the GeneAmp^®^ PCR system 9700 (Applied Biosystems, CA, USA). qPCR analyses were conducted by using SYBR Green PCR master mix (Takara, Tokyo, Japan) in the 7500 RT‐PCR system (Applied Biosystems, Thermo Fisher Scientific, Waltham, MA, USA). Glyceraldehyde 3‐phosphate dehydrogenase (GAPDH) (B661304, Sangon Biotech, Shanghai, China) was used as an internal control. Primer sequences (Sangon Biotech, Shanghai, China) for mouse primers are as follows: Mmp13 forward, 5'‐CCAGAACTTCCCAACCATGT‐3' and reverse, 5'‐GTCTTCCCCGTGTTCTCAAA‐3'; Col1‐α1 forward, 5'‐AGAACATCACCTACCACTGCAAGA‐3' and reverse, 5'‐GTTTTGTATTCAATGATTGTCTTGC‐3'; CTGF: 5’‐CCGCCAACCGCAAGATT‐3’, and reverse: 5’‐ACCGACCCACCGAAGACA‐3’; Tgfb1 forward, 5'‐ GTGGAAATCAACGGGATCAG‐3’ and reverse, 5’‐ ACTTCCAACCCAGGTCCTTC‐3’; Tgfb3 forward, 5'‐CCAAATCAGCCTCTCTCTGT‐3' and reverse, 5’‐AATGGCTTCCACCCTCTTC‐3’; GAPDH: forward, 5’‐ACTCCACTCACGGCAAATTC‐3’ and reverse 5’‐TCTCCATGGTGGTGAAGACA‐3’. Primer sequences for human primers are as follows: Col1‐α1 Sense: 5'‐GGCTTCCCTGGTCTTCCTGG‐3', Antisense: 5'‐CCAGGGGGTCCAGCCAAT‐3'; α‐SMA Sense: 5'‐AGGCACCCCTGAACCCCAA‐3', Antisense: 5'‐CAGCACCGCCTGGATAGCC‐3'; GAPDH; Sense: 5’‐GGTGAAGGTCGGAGTCAACGG‐3’, Antisense: 5’‐TGAAGGGGTCATTGATGGCAACA‐3’. The mRNA expression levels of the detected genes were normalized to that of GAPDH.

### 2.8 Western Blot

LX‐2 cell extracts were prepared on ice with lysis buffer containing 1 mM phenylmethylsulfonyl fluoride (PMSF) (Beyotime, Shanghai, China). Protein concentration was determined with a Bio‐Rad DC kit (Bio‐Rad). Equal amounts of protein were separated by 8% SDS‐PAGE and transferred onto polyvinylidene difluoride (PVDF) membranes (Millipore Corp., Billerica, MA, USA). The membranes were blocked with 5% non‐fat milk in TBST buffer for 1 hour at room temperature and then incubated overnight at 4°C with the following primary antibodies: collagen I (Abcam, Cambridge, MA, USA), α‐SMA (Abcam) and GAPDH (Santa Cruz, Santa Cruz, USA), and incubated with secondary horseradish peroxidase(HRP)‐conjugated antibodies (Yeasen, Shanghai, China) for 1 hour at room temperature. The signals were visualized by enhanced chemiluminescence (ECL) method using chemiscope 5600 (CLINX, Shanghai, China).

### 2.9 Construction of positive feedback loop between Tgbf3 and Mmp13 on collagen accumulation

Tgfb3 and Mmp13 have been found to inhibit each other based on the observed data as well as the available interaction information (Supplemental Figure S1). We next constructed the following dimensionless model for the network of Tgfb3 and Mmp13 similar to the toggle model: ^24^, ^25^

$$\frac{dx(t)}{\mathrm{dt}}=\frac{\alpha_{1}}{1+y{\left(t\right)}^{\beta_{1}}}‐x\left(t\right).$$

$$\frac{dy(t)}{\mathrm{dt}}=\frac{\alpha_{2}}{1+x{\left(t\right)}^{\beta_{2}}}‐y\left(t\right).$$

Here, *x* and *y* represent the mean expression level of Tgfb3 and Mmp13, respectively. $\alpha_{1}$ and $\alpha_{2}$ are the effective rate of synthesis for Tgfb3 and Mmp13, while $\beta_{1}$ and $\beta_{2}$ are the cooperativity factors. Obviously, it is a positive feedback loop as a result of two consecutive negative interactions. Phase diagram was built by solving the above equations, and clearly bistability occurred (Figure 6A2 and 6A3). The parameters *α* and *β* were determined by appropriate values based on the ranges of available data and simulation.

## 3 RESULTS

### 3.1 Animal model of liver fibrosis

Images of H&E, Masson's trichrome and reticulin stained samples for assessing the stage of liver fibrosis are shown in Figure 1. The TAA‐treated mice first developed liver injuries leading to mild, then significant fibrosis and eventually liver cirrhosis. Compared with the control mouse liver with normal hepatic architecture, the liver tissues from mice treated with TAA demonstrated marked cell necrosis, inflammatory cell infiltration, and fibrous septa formation. As judged by conventional histological descriptions and histological fibrotic classification scores,^26^ the histological clarification of mice with TAA treatment for 3 and 5 weeks is similar to METAVIR stage 1 (portal fibrosis) and stage 2 (portal fibrosis with septae outside the portal area), respectively; week 9 as stage 3, bridging fibrosis; weeks 14 and 17 as stage 4, cirrhosis, showing complete fibrous septa formation surrounding pseudolobuli.

The pathological feature of week 9 TAA treatment samples is significant in the increased distribution and amount of fibrotic tissues than in samples from former time‐points.

### 3.2 Gene expression profiling

**FIGURE 2:**
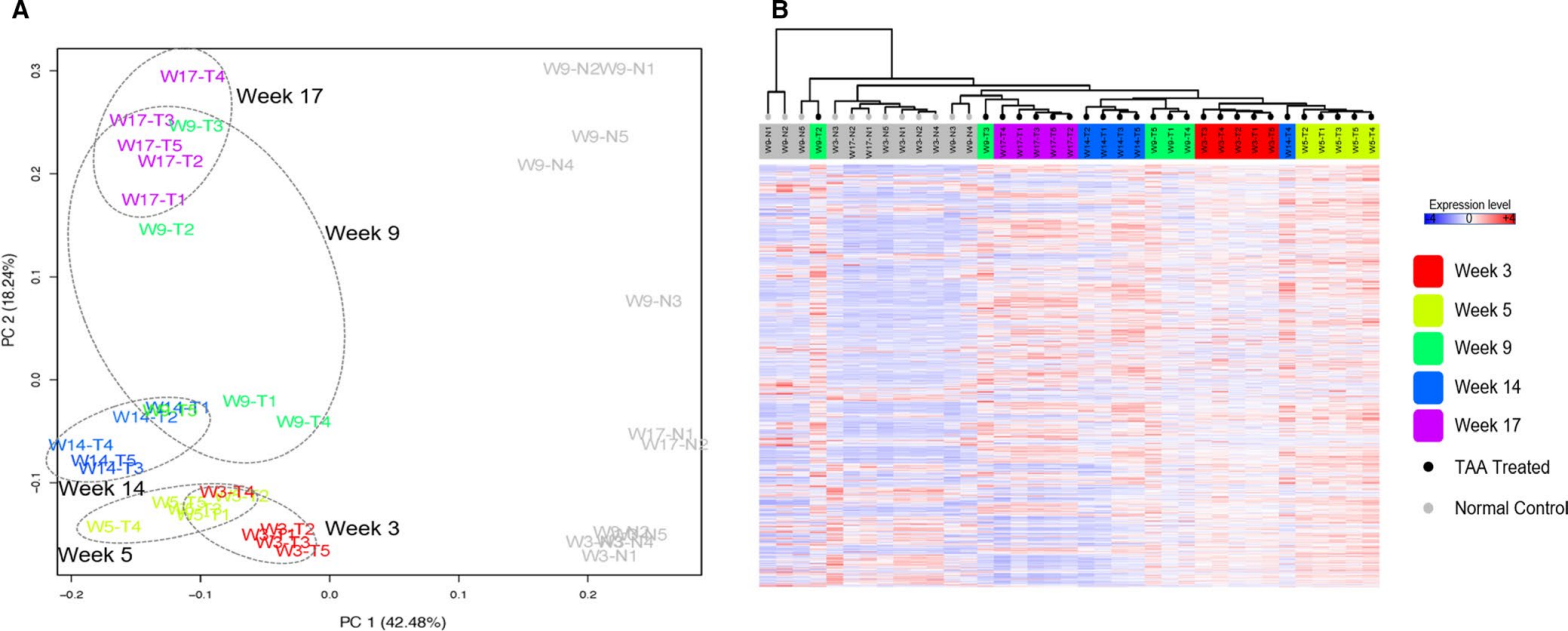
Differentially expressed gene (DEG) analysis of the time series of liver transcriptomes. (A) Principle component analysis (PCA) on DEGs of the time series of liver transcriptomes showed that DEGs of the livers from mice with TAA treatment for week 9 samples were not clustered together but dispersed, whereas samples from mice collected at the other time‐points post‐TAA treatment were clustered well together. (B) Unsupervised hierarchical analysis also showed that week 9 samples were unique and cannot be clustered together

After multiple comparisons, 3602 differentially expressed genes (DEGs) were identified with adjusted *P*‐value <0.05. By principle component analysis (PCA) and unsupervised hierarchical analysis (HCA) on DEGs, different stages of liver injuries were distinguished. Samples from mice with TAA treatment for 9 weeks were not clustered together but dispersed (Figure 2A), whereas sample groups collected at the other time‐points were clustered together. Similar to PCA, HCA (Figure 2B) showed that samples of TAA‐treated mice for 9 weeks cannot be clustered together, whereas samples collected at the other time‐points can. The result implied that week 9 was unique and different from the other time‐points. It may represent a critical state or tipping point for significant fibrosis transition, which was in line with the histological finding with H&E, Masson's trichrome and reticulin staining for fibrotic tissues.

### 3.3 DNB analysis identifies the tipping point just before liver fibrosis transition

**FIGURE 3:**
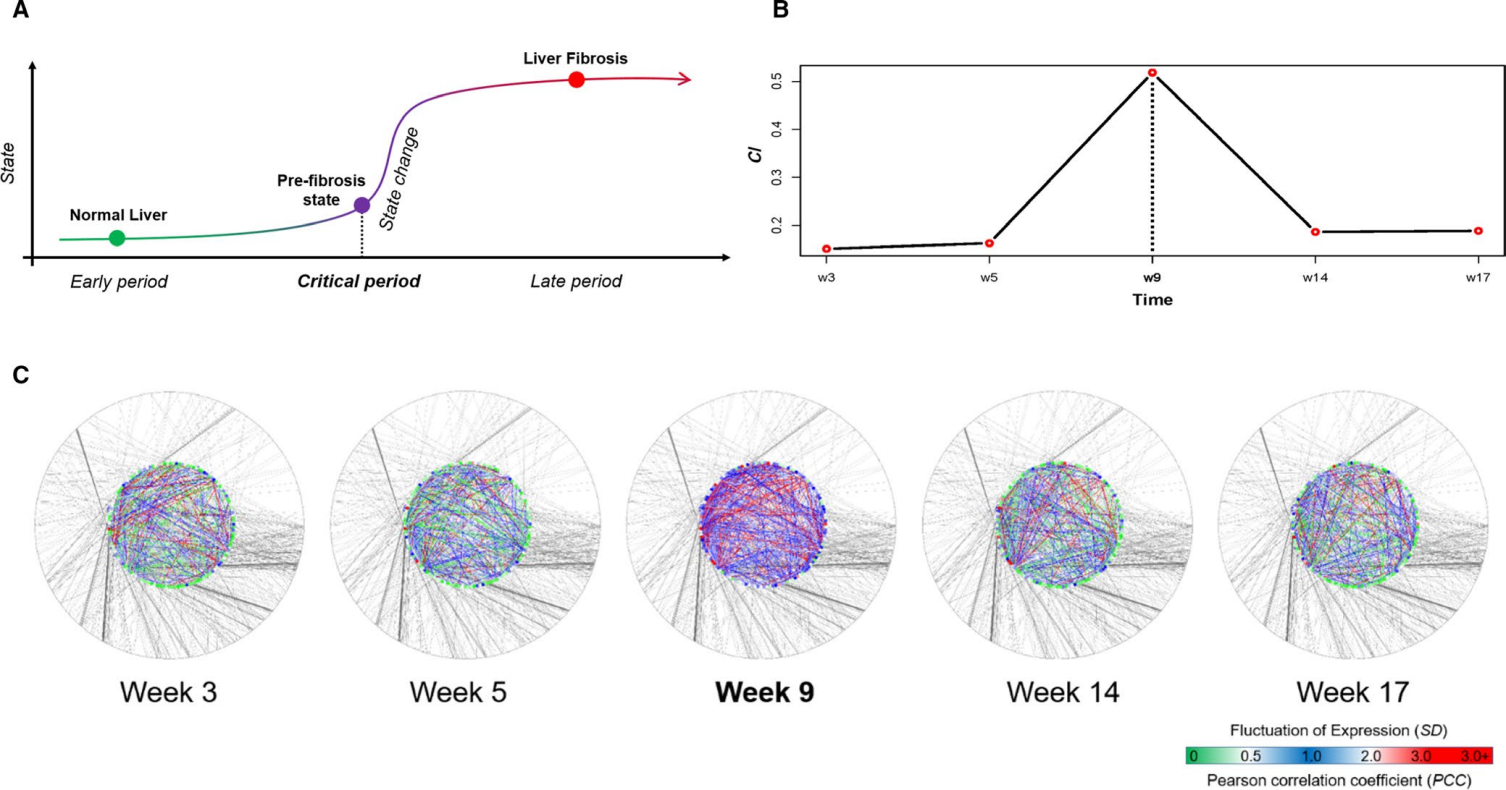
Dynamic network biomarkers (DNB) analysis identified the tipping point of liver fibrosis transition. (A) Based on DNB theory, the progression of liver fibrosis was divided into three phases as follows: normal state, pre‐fibrotic state (or critical state) and fibrosis state. (B) DNB analysis on the time series of liver transcriptome data of mice with TAA‐induced liver fibrosis. A strong signal indicated the critical state was found at week 9 by calculating the composite index (CI). (C)The DNB‐associated network was obtained by integrating knowledge‐based molecular interactions. Different colours were mapped to nodes and links according to the adjusted standard deviations (SDs) of each gene and the adjusted Pearson's correlation coefficients (PCCs) of pair‐wise genes, respectively. The adjusted values (SDs and PCCs) were calculated by the original values of TAA‐treated mice compared with the average values of all normal control mice

Based on the DNB theories,^10^, ^11^, ^27^ the progression of a specific disease was divided into three phases as follows: normal, pre‐disease (tipping point or critical state) and disease states (Figure 3A). To accurately determine the critical stage of fibrosis progression in TAA‐induced animal model of liver fibrosis, we applied DNB analysis on the time series of liver transcriptome data of mice with TAA‐induced liver injuries. A strong signal of the critical state was found at week 9 as calculated using CI (Figure 3B), indicating that week 9 was a critical time‐point of phase transition from mild to significant liver fibrosis. This result was consistent with both the genes in the differential expression analysis and histopathological features.

A set of DNB genes containing 153 genes were obtained by the selection criteria of DNB members for further analysis. As DNBs were considered leading factors with critical functions in disease‐associated progression of complex diseases, the mechanism of these DNBs in fibrogenesis at a network level was analysed (Figure 3C). The DNB‐associated network was obtained by integrating knowledge‐based molecular interactions from BioGRID,^28^ StringDB,^29^ AnimalTFDB^30^ and RegNetwork.^31^ Different colours were mapped to nodes and links according to the adjusted *SD*s of each gene and the adjusted *PCC*s of pair‐wise genes, respectively. The adjusted values (*SD*s and *PCC*s) were calculated by the original values of TAA‐treated mice compared with the average values of all normal control mice. As shown in Figure 3C, a clear signal for DNBs was found at week 9, similar to the calculated *CI* result Figure 3B. Note that there is still no significant signal based on traditional DEG analysis at week 9, which confirmed the effectiveness of DNB as the early warning signals.

### 3.4 Functional analysis revealed that fibrosis‐associated pathways are influenced by DNBs

**FIGURE 4:**
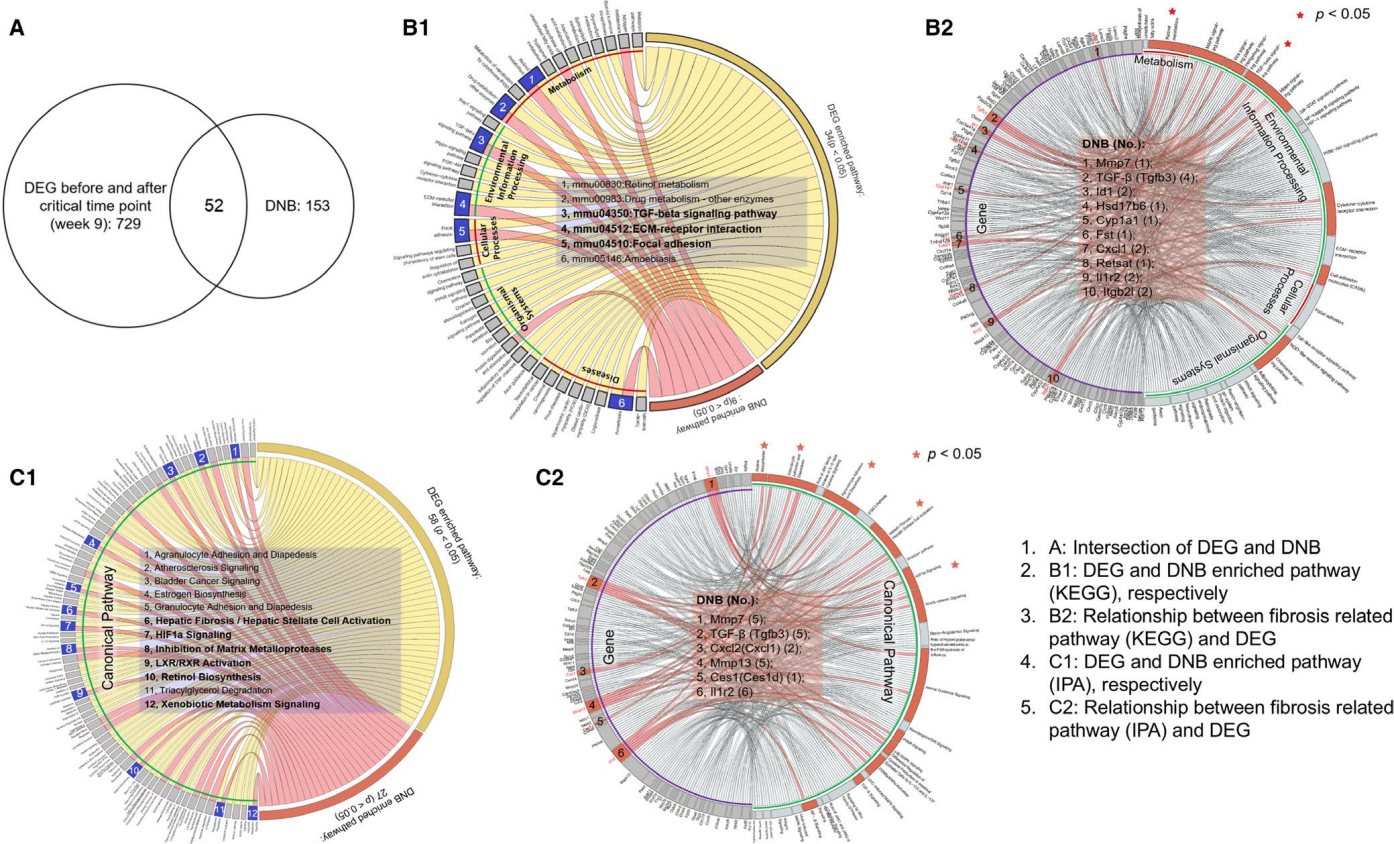
Functional analysis revealed that fibrosis‐associated pathways were influenced by a subset of DNBs. (A) Differentially expressed genes (DEGs) were calculated before and after week 9 using DEseq2. Fifty‐two genes were identified to be both DEGs and DNBs. (B) Functional analyses were performed on DEGs and DNBs separately. KEGG and IPA databases were used for pathway detection. All significant DEGs and DNBs enriched canonical pathways from KEGG (B1) and IPA databases (C1), and the DEGs and DNBs enriched significant fibrosis‐associated pathways from KEGG (B2) and IPA (C2). DEGs were marked on the left side, while fibrosis‐associated pathways were marked on the right of the figures. Pink colour linked the specific DEG to DNBs with the corresponding pathways that the DEG was a key molecule. Fibrosis‐associated signalling pathways noted in B1 includes mmu00830 (Retinol metabolism), mmu04350 (TGF‐β signalling pathway), mmu04512 (ECM‐receptor interaction) and mmu04510 (focal adhesion). Fibrosis‐associated signalling pathways noted in C1 include hepatic fibrosis/Hepatic stellate cell activation, HIF1α signalling, inhibition of matrix metalloproteases, LXR/RXR activation and retinol Biosynthesis. DNB (number of pathways associated with this gene) noted in B2 includes: 1. Mmp7 (1); 2. TGF‐β (Tgfb3) (4); 3. Id1 (2); 4. Hsd17b6 (1); 5. Cyp1a1 (1); 6. Fst (1); 7. Cxcl1 (2); 8. Retsat (1); 9. Il1r2 (2); 10. Itgb21 (2). DNB (number of pathways associated with this gene) noted in C2 includes 1. Mmp7 (5); 2. TGF‐β (Tgfb3) (5); 3. Cxcl2 (Cxcl1) (2); 4. Mmp13 (5); 5. Ces1 (Ces1d) (1); 6. Il1r2 (6)

As week 9 in the experimental model of TAA‐induced liver fibrosis was determined as the tipping point just before significant fibrosis transition, DEGs were calculated before and after week 9 using DEseq2. 729 differentially expressed genes were identified and 52 of them existed in DNBs (Figure 4A, Supplemental Table S1). Functional analyses were then performed on DEGs and DNBs separately. Two databases (ie KEGG and IPA) were used for canonical pathway detection. All significant DEGs and DNBs enriched pathways from KEGG and IPA databases are shown in Figure 4B1 and 4C1, respectively. In the six common KEGG pathways in which both DEGs and DNBs were enriched, four were associated with fibrosis (ie mmu00830, retinol metabolism; mmu04350, TGF‐β signalling pathway; mmu04512, ECM‐receptor interaction; mmu04510, focal adhesion) (Figure 4B1). Similar to KEGG, most of the common enriched IPA pathways of DEGs and DNBs were associated with fibrosis, including hepatic fibrosis/HSC activation, HIF1α signalling, inhibition of matrix metalloproteases (MMP), LXR/RXR activation and retinol biosynthesis (Figure 4C1). The result further confirmed our previous results pointing to week 9 as the critical period for significant fibrosis transition.

Moreover, we collected all potential fibrosis‐associated pathways based on previous publications in two databases (Supplemental Tables S2, S3). The existence of DEGs and DNBs in each of these pathways was detected. The resulting DEGs enriched significant KEGG and IPA pathways are shown in Figure 4B2 and Figure 4C2, respectively. DEGs are marked on the left side, while fibrosis‐associated pathways were marked on the right. Pink colour linked the specific DEG to DNBs with the corresponding pathways in which DEG is a key molecule. From the result based on the KEGG database, 10 DNBs (Mmp7, Tgfb3, Id1, Hsd17b6, Cyp1a1, Fst, Cxcl1, Retsat, Il1r2, Itgb21) were found and considered key fibrosis‐associated genes. The number followed by the DNB gene symbol in the figure represents how many pathways are associated with this gene. Similarly, we identified six key DNBs by IPA database, including Mmp7, TGF‐β (Tgfb3), Cxcl2 (Cxcl1), Mmp13, Ces1 (Ces1d) and Il1r2. Symbols before parentheses are IPA conversion of the input identifier (ID). The conversions are important paralog with biological function and molecular correspondence. Most of them were the same as the DNBs listed in fibrosis‐related KEGG pathways. The combination of both DNB lists resulted in a total number of 12 DNBs as candidate critical genes. GO annotations related to this gene and related pathways are listed in Supplemental Table S4.

### 3.5 Tgfb3 plays a key role in the progression of liver fibrosis based on DNB ranking

**FIGURE 5:**
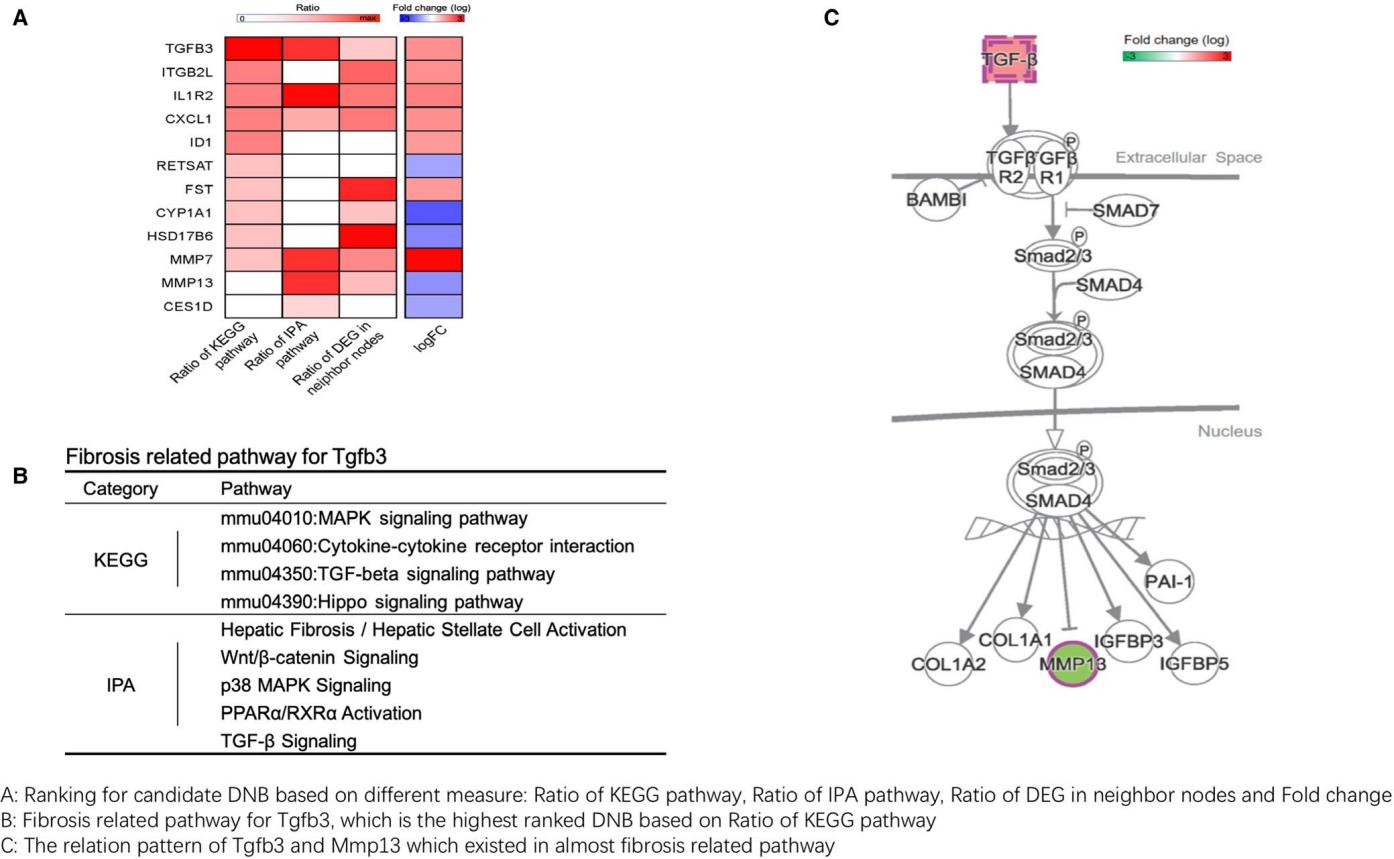
DNB ranking and functional analysis for the liver fibrosis progression. (A) Twelve candidate DNB genes were ranked based on the total number of their involved fibrosis‐associated KEGG or IPA‐annotated pathways. Tgfb3 was the top and up‐regulated DNB gene based on the ranking. Both DNB genes and DEGs before and after week 9 were mapped into a pre‐combined network (combination of BioGRID, StringDB, AnimalTFDB and RegNetwork), and the total number of DEGs that directly linked with each DNB gene was individually counted. Each DNB was also marked for whether it is up‐regulated after the critical state (week 9) or not. (B) Fibrosis‐related pathways from KEGG and IPA for Tgfb3. (C) Extracted paths starting with Mmp13 and ending with Tgfb3 from the interaction networks in IPA

To uncover which of the 12 candidate DNBs was more important in fibrosis progression, we ranked the DNB genes based on the total number of their involved fibrosis‐associated KEGG or IPA‐annotated pathways. In addition, we mapped both DNB genes and DEGs before and after week 9 into a pre‐combined network (combination of BioGRID, StringDB, AnimalTFDB and RegNetwork) and individually counted the total number of DEGs directly linked with each DNB gene. We also marked each DNB for whether it is up‐regulated after the critical time‐point (week 9). The results are shown in Figure 5A. Tgfb3 ranked first, followed by up‐regulated DNB. This gene encoded TGF‐β3, a cytokine belongs to TGF‐β superfamily that is involved in ECM formation and wound healing.^32^

### 3.6 Functional analysis of Tgfb3 on fibrogenic gene expression in HSCs

Mmp13 was also found to be a key DNB for the critical transition of fibrogenesis. This gene encoded collagenase 3 that is known to play a role in ECM protein degradation.^33^, ^34^ To further delineate the interaction within Tgfb3 and Mmp13, and type I collagen that is the main substrate of Mmp13, all fibrosis‐related pathways from KEGG and IPA for Tgfb3 were collected (Figure 5B) for analysing pathway‐based interaction between them (Figure 5C). By searching the whole interaction networks in IPA, all paths that started with Mmp13 and ended with Tgfb3 were extracted. As shown in Supplemental Figure S1 for these sub‐networks, Tgfb3 negatively regulated Mmp13 via activating Smad2 and Smad3.

**FIGURE 6:**
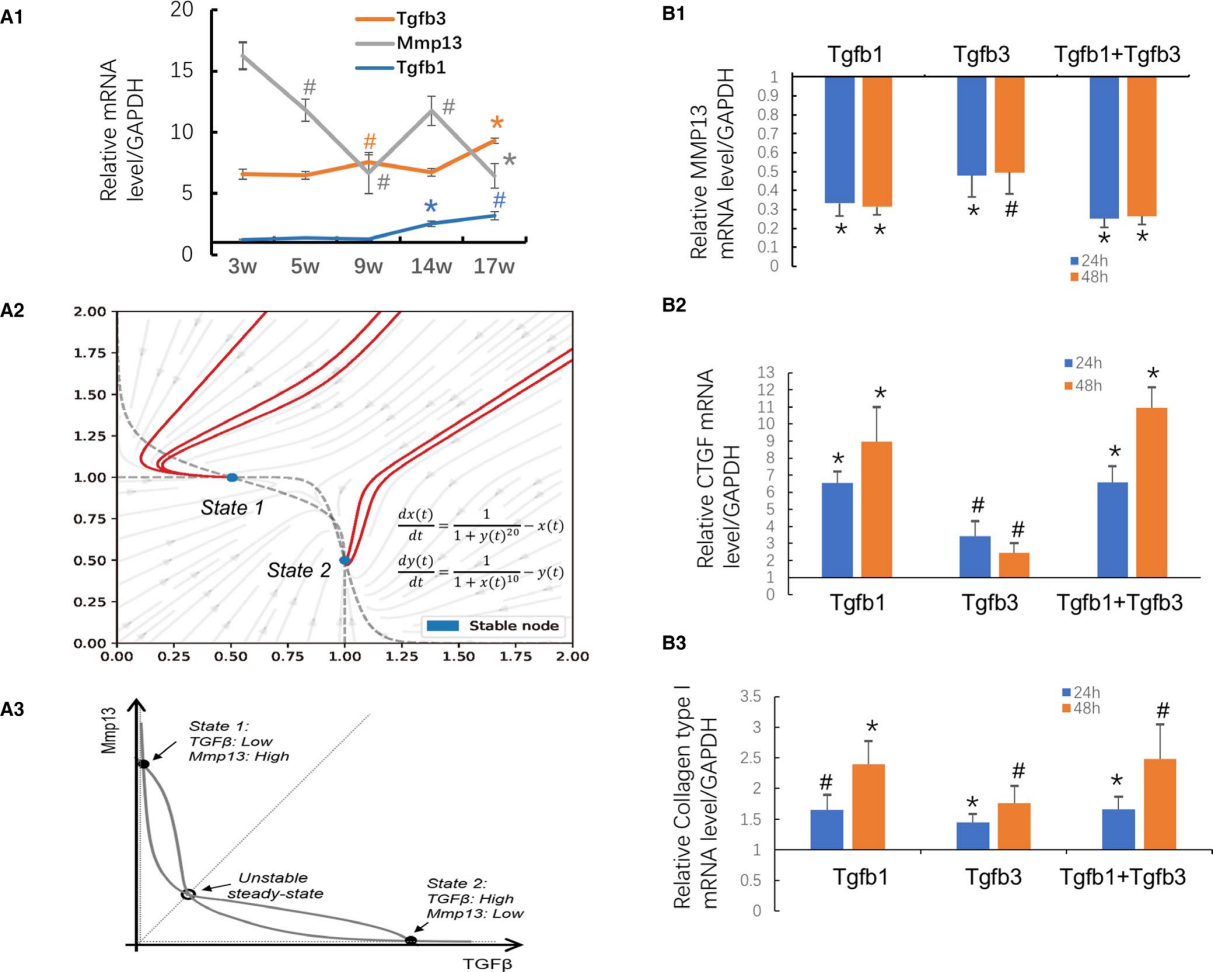
Expression patterns of Tgfb3, Tgfb1 and Mmp13 in TAA‐induced liver fibrosis and functional analyses of Tgfb3 on fibrogenic gene expression in hepatic stellate cells. (A1) Real‐time quantitative polymerase chain reaction (RT‐qPCR) analyses of the transcription levels of Tgfb3, Tgfb1 and Mmp13 in the time series of liver samples of mice with TAA‐induced liver injury and fibrosis. ^*^ *P* <0.01, ^#^ *P* < 0.05compared with the adjacent prior time‐point. (A2) Phase map of the regulation between Tgfb3 (*x*) and Mmp13 (*y*) transcription in the liver during fibrogenic process. (A3) Dynamics of the bistable model for Tgfb3 and Mmp13 transcription in the liver during fibrogenic process. (B)Effects of Tgfb3 and/or Tgfb1 onMMP13 (B1), CTGF (B2) and collagen type I (B3) genes expression in hepatic stellate cell line JS1. Data are presented as mean ± SD of N = 4 per group in three independent experiments. ^*^ *P* <0.01, ^#^ *P* <0.05 compared with cells treated with saline vehicle

RT‐qPCR analyses revealed that the Tgfb3 expression in the liver of TAA‐treated mice increased after TAA administration and first peak at 9 weeks, whereas the increase of Tgfb1 expression was found to lag behind Tgfb3 and peaked at 17 weeks post‐TAA administration (Figure 6A1).

The functional influence of Tgfb3 on fibrogenic gene (eg Mmp13, collagen type 1, CTGF) expression was further investigated by an *in vitro* study with an immortalized mouse HSC line JS1. As shown in Figure 6B, Tgfb3 time dependently promotes the fibrogenic (eg collagen type 1, CTGF) gene expression, but JS1 cells down‐regulate Mmp13 gene expression. The regulatory effects of Tgfb3 on collagen type 1, CTGF and Mmp13 gene expression are similar to those of its isoform Tgfb1. Similarly, a promoting effect of Tgfb3 on collagen type I and SMA mRNA expression and protein levels was also found in human HSC line LX2 (Supplemental Figure S2).

### 3.7 Bistable model of Tgfb3 and Mmp13 during fibrotic process

Based on the theoretical model for network, the phase map on the regulation between Tgfb3 and Mmp13 during fibrogenic process was generated, which is clearly a bistable system from a viewpoint of dynamics (Figure 6A2, Supplemental Figure S3). The steady (stable) state 1 is the normal stage with relatively higher expression of Mmp13 and lower expression of Tgbf3 (Figure 6A3), so that collagen can be removed properly. When environmental burden (ie TAA stimulation) exists, the production of Tgfb3 increases and collagen accumulates as a scar repairing response. As the injury further continues, the production of Tgfb3 would pass the tipping point (near unstable steady state) and eventually reach another stable state (steady state 2) (Figure 6A3), where the expression and activity of Mmp13 inversely decrease and the fibrillar collagen would be unremovable.

## 4 DISCUSSION

In the present study, DNB analysis was applied to our time series of liver transcriptomes of mice with TAA‐induced liver fibrosis to identify the critical state or tipping point during liver fibrogenesis process, which was found to be at week 9, pathologically relevant to METAVIR stage 3, that is bridging fibrosis, with a set of DNB genes that may exert critical function driven by significant activation of fibrogenic signalling pathways at a network level. This result was in line with a previous report that an increase in METAVIR stage is associated with a progressive increase in the fibrosis area and the increase of fibrous tissue accumulation is not linear.^35^ Less reversibility was found in late stages of fibrosis as evidences obtained from experimental studies and clinically liver biopsied patients.^36^, ^37^ Significant increase and accumulation of fibrotic tissue happens in stage 3 during fibrogenesis. The treatment of high fibrosis staging requires ‘dissolution’ of more fibrous tissue; hence, it is more difficult. The results indicated that clinically reliable diagnosis before stage 3 and early intervention are important to achieve a complete fibrosis reversion.

In this study, 153 DNB genes were obtained, of which 52 were DEGs. The GO and pathway analysis revealed that the 52 differentially expressed DNBs are enriched in known important fibrosis‐associated pathways, such as retinol metabolism, TGF‐β signalling pathway, ECM‐receptor interaction, focal adhesion, hepatic fibrosis/HSC activation, HIF1α signalling, MMP inhibition, LXR/RXR activation and retinol biosynthesis. These pathways are known key signalling pathways involved in the initiation and maintenance of HSC activation and increased synthesis of ECM and decreased ECM degradation. Dynamics of liver pathologies in liver fibrosis progression can be described by the DNB gene expression profile. Their differential expressions before and after week 9 coincident with significant fibrosis progression further indicate a systemic transition point and global activation of fibrogenic signalling pathways at around week 9 in this model, when these DNBs participate in the activation of the fibrogenic cascade in a network manner. The result further confirmed the theory that DNB members are more likely to be ‘drivers’ or causal genes of diseases^9^, ^15^; thus, in this specific fibrogenesis setting, interventions targeting these DNBs at this critical stage may alter the fibrosis progression. Moreover, as DNBs correspond to pre‐disease stage or disease onset time‐point before drastic deterioration, they can be directly used for early disease diagnosis or predictive medicine. The DNBs identified in this study warrant further verification on their usage as fibrosis treatment targets and diagnostic markers.

The analysis of differentially expressed DNBs that are enriched in significant fibrosis‐associated KEGG and IPA pathways revealed 12 DNBs (Mmp7, TGF‐β (Tgfb3), Id1, Hsd17b6, Cyp1a1, follistatin (FST), Cxcl2 (Cxcl1), Retsat, Il1r2, Itgb21, Mmp13 and Ces1 (Ces1d) as key fibrosis‐associated genes. Among them, Tgfb3, FST and Id1 are involved in TGF‐β signalling, which is the post‐potent fibrogenic pathway with downstream products including CTGF and collagen type I. Ligands of this family bind various TGF‐β receptors, leading to recruitment and activation of Smad family transcription factors that regulate gene expression. Id1 has been identified to be TGF‐β/ALK1/Smad1 target gene in HSCs and is a critical mediator in TGF‐β induced HSC transdifferentiation.^38^ Mmp13 and Mmp7 are key members of MMPs (also called matrixins) family. They are zinc‐dependent endopeptidases and major proteases in ECM protein degradation. Mmp13 cleaves triple helical collagens, including type I, II and III collagens, and fibronectin. It may also function by activating or degrading key regulatory proteins, such as Tgfb1 and CTGF.^39^, ^40^, ^41^ Mmp7 is also a secreted protease that degrades proteoglycans, fibronectin, elastin, casein, gelatins and fibronectin. During liver fibrogenesis, the degradation of ECM by MMPs fails to keep pace with increased synthesis, in part as a result of sustained expression of MMP inhibitors (eg tissue inhibitors of metalloproteinases).^42^ Integrin subunit beta 2 encodes an integrin beta chain, which combines with multiple different alpha chains to form different integrin heterodimers. Integrins are integral cell surface proteins that participate in cell adhesion as well as cell surface‐mediated signalling. Integrins on myofibroblasts are components of a core cellular and molecular pathway that contributes to pathologic fibrosis in multiple solid organs.^43^ IL1R2 and Cxcl1 are involved in immune and inflammation response. Cyp1a1 catalyses many reactions involved in drug metabolism and synthesis of cholesterol, steroids and other lipids involved in the detoxification of xenobiotics. Retsat, Ces1 (Ces1d) and HSD17B6 are enzymes that are involved in the metabolism of vitamin A retinol conversion. The association of these genes with liver fibrosis and the effect of blocking them as antifibrotic targets warrant further investigation.

The ranking result of the DNB genes based on the total number of their involved fibrosis‐associated KEGG or IPA‐annotated pathways revealed that Tgfb3 was at the top and up‐regulated DNB gene. TGF‐β is a pleiotropic cytokine with various effects on cellular behaviour including proliferation, migration, invasion, angiogenesis and immune responsiveness. Three TGF‐β isoforms, Tgfb1, Tgfb2, Tgfb3, exhibit high levels of similarity in their amino acid sequences. Tgfb3 signalling pathway mediated by Tgfb3 controls many cellular functions during embryogenesis, including differentiation, proliferation and migration.^44^, ^45^ As Tgfb1, Tgfb3 signals through the assembling receptor complex and phosphorylation‐dependent activation of Smad transcription factors and downstream signalling cascades.^46^, ^47^, ^48^, ^49^ Both are important mediators of fibrogenesis within the course of chronic hepatic allograft rejection^50^ and attributed a key role in the pathogenesis of glioblastoma.^47^ Up‐regulation of Tgfb3 expression was found in cirrhotic livers of primary biliary cirrhosis patients^51^ and in CCl4‐treated rat livers.^52^ Higher Tgfb3 level in patients without non‐alcoholic fatty liver diseases (NAFLD) was associated with higher chance of future development of NAFLD.^53^ In Con A‐induced autoimmune liver‐damaged mice, abnormal HSC activation was accompanied by imbalance of Tgfb1 and Tgfb3 expressions in the early stage, which can line immune and hepatic fibrosis processes, leading to further switching and progressing of liver fibrosis.^54^ In this study, both Tgfb3 and Tgfb1 were found to inhibit Mmp13 expression in HSC *in vitro*. The expression of Tgfb3 increased earlier than Tgfb1 *in vivo* in the liver of mice with TAA‐induced experimental liver fibrosis, suggesting that Tgfb3 may be more important than Tgfb1 in initiating fibrogenesis.

Mmp13 is a zinc‐containing interstitial collagenase capable of degrading native fibrillar collagens and plays a key role in the remodelling of connective tissue matrix under both physiological and pathological conditions.^55^, ^56^ In normal and fibrotic livers, expression of collagen types I, III and IV takes place predominantly in non‐parenchymal cells.^57^ During early fibrogenesis, the hepatic fibrillar collagens are first cleaved by Mmp13, and the partially denatured collagens are subsequently digested by gelatinolytic activities of other MMPs.^58^ Mmp13 is involved in the degradation of hepatic ECM in the initial phase of hepatic fibrosis and pave the way for the proliferation of activated HSC that transforms into myofibroblasts. In this study, a bistable model of Tgfb3 and Mmp13 during fibrogenic process was constructed, and the analysis indicates that these two DNBs can suppress each other by forming the positive feedback loop and exhibit the bistable dynamics, which implies the molecular mechanism of the switching behaviour. Increased Tgfb3 expression in liver fibrogenesis and its inhibitory effect on Mmp13 expression suggested an earlier contribution of Tgfb3 to a ceaseless accumulation of collagens by inhibition of Mmp13 expression, resulting in significant fibrogenesis from the tipping point on. This was in line with clinical profiles of the expression of Tgfb3 and MMP13 in the liver of cirrhotic patients.^50^, ^53^, ^56^, ^59^, ^60^, ^61^ Early intervention of Tgfb3 or both Tgf3 and Tgfb1 may be required for antifibrotic therapies.

In conclusion, liver fibrosis is driven by many related genes to progress from liver damage to significant fibrotic stages. A set of DNBs that may exert critical function drives the activation of fibrogenic signalling pathways in a network manner. Fibrosis stage 3 relevant to bridging fibrosis is a critical point for significant fibrosis transition. Key factors for the critical transition to significant fibrosis as a result of ceaseless accumulation of collagens include Mmp7, TGF‐β (Tgfb3), Id1, Hsd17b6, Cyp1a1, Fst, Cxcl2 (Cxcl1), Retsat, Il1r2, Itgb21, Mmp13 and Ces1 (Ces1d). The results emphasize that identifying fibrosis prior to stage 3 and treating patients is important for fibrosis reversion, and this study may provide novel targets for antifibrotic therapies, specifically Tgfb3 and other key DNBs. Although we have identified the tipping point as well as a potentially important function of Tgfb3 in TAA‐induced mice model of liver fibrosis, the results warrant further validation in other animal models or in patients with chronic liver diseases. Mechanism studies are also important in order to elucidate the impact of Tgfb3 on such a critical transition near the tipping point.

## CONFLICTS OF INTEREST

The authors declare no conflict of interest.

## AUTHOR CONTRIBUTIONS

**Jinsheng Guo:** Conceptualization (lead); Data curation (lead); Formal analysis (lead); Funding acquisition (equal); Investigation (lead); Methodology (lead); Resources (lead); Software (equal); Supervision (lead); Validation (equal); Visualization (equal); Writing‐original draft (equal); Writing‐review & editing (lead). **Weixin Liu:** Formal analysis (lead); Investigation (equal); Methodology (equal); Software (lead); Writing‐original draft (equal). **Zhiping Zeng:** Investigation (equal). **Jie Lin:** Formal analysis (equal); Software (equal). **Xingxin Zhang:** Investigation (equal). **Luonan Chen:** Conceptualization (lead); Data curation (equal); Formal analysis (equal); Funding acquisition (lead); Investigation (equal); Methodology (supporting); Resources (equal); Software (lead); Supervision (lead); Validation (equal); Visualization (equal); Writing‐original draft (equal); Writing‐review & editing (equal).

## Supporting information

Fig S1

Fig S2

Fig S3

Table S1‐S4

## Data Availability

All the citations and data included in this manuscript are available upon request by contact with the corresponding authors.
