# Supplementary figures and images for: Tgfb3 and Mmp13 regulated the initiation of liver fibrosis progression as dynamic network biomarkers
Source: J Cell Mol Med. 2020 Dec 2;25(2):867–79. doi: 10.1111/jcmm.16140 (PMC7812286; doi:10.1111/jcmm.16140)

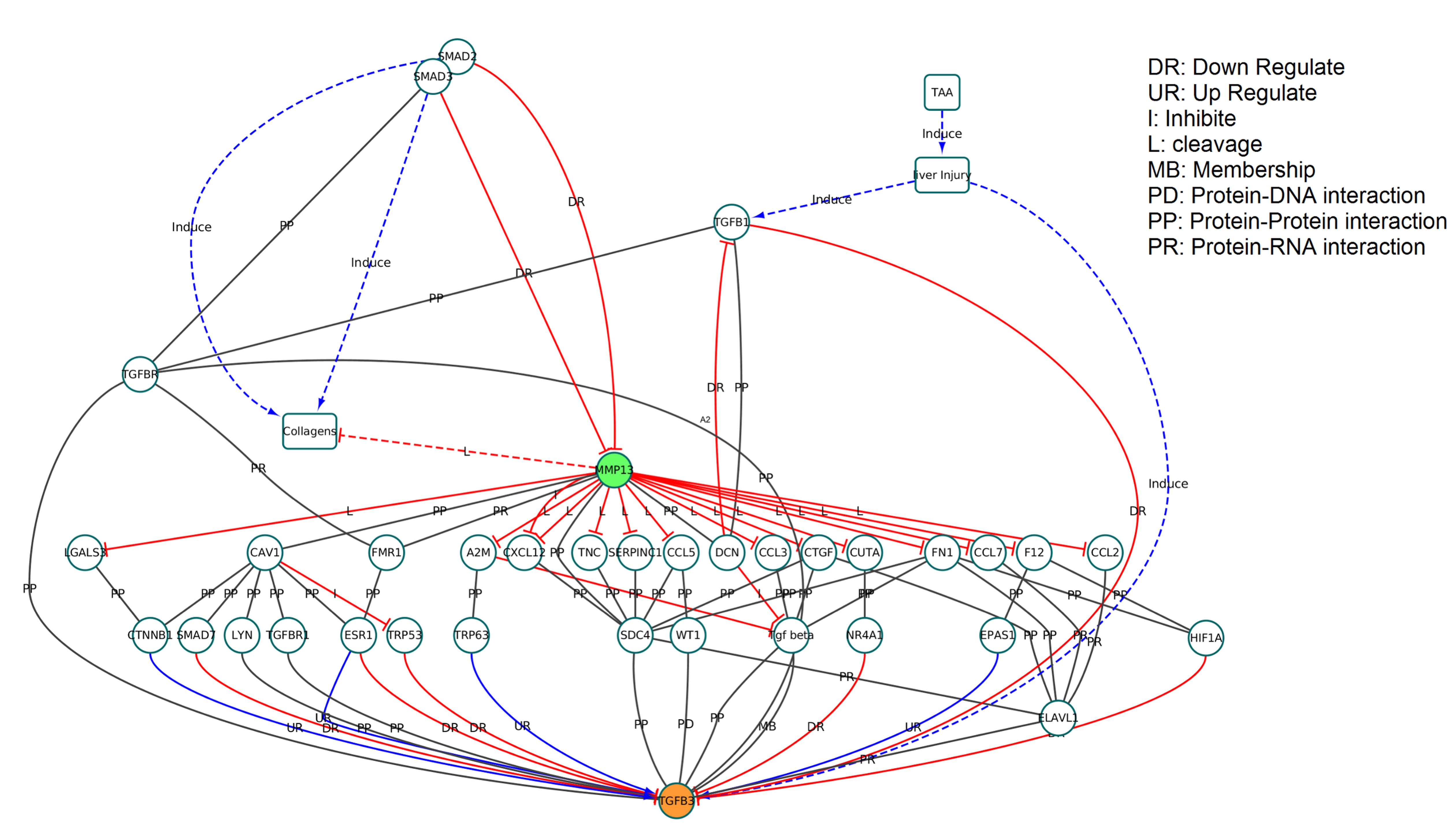

Supplement: Supplementary file 1 — Fig S1 [file JCMM-25-867-s001.jpg]

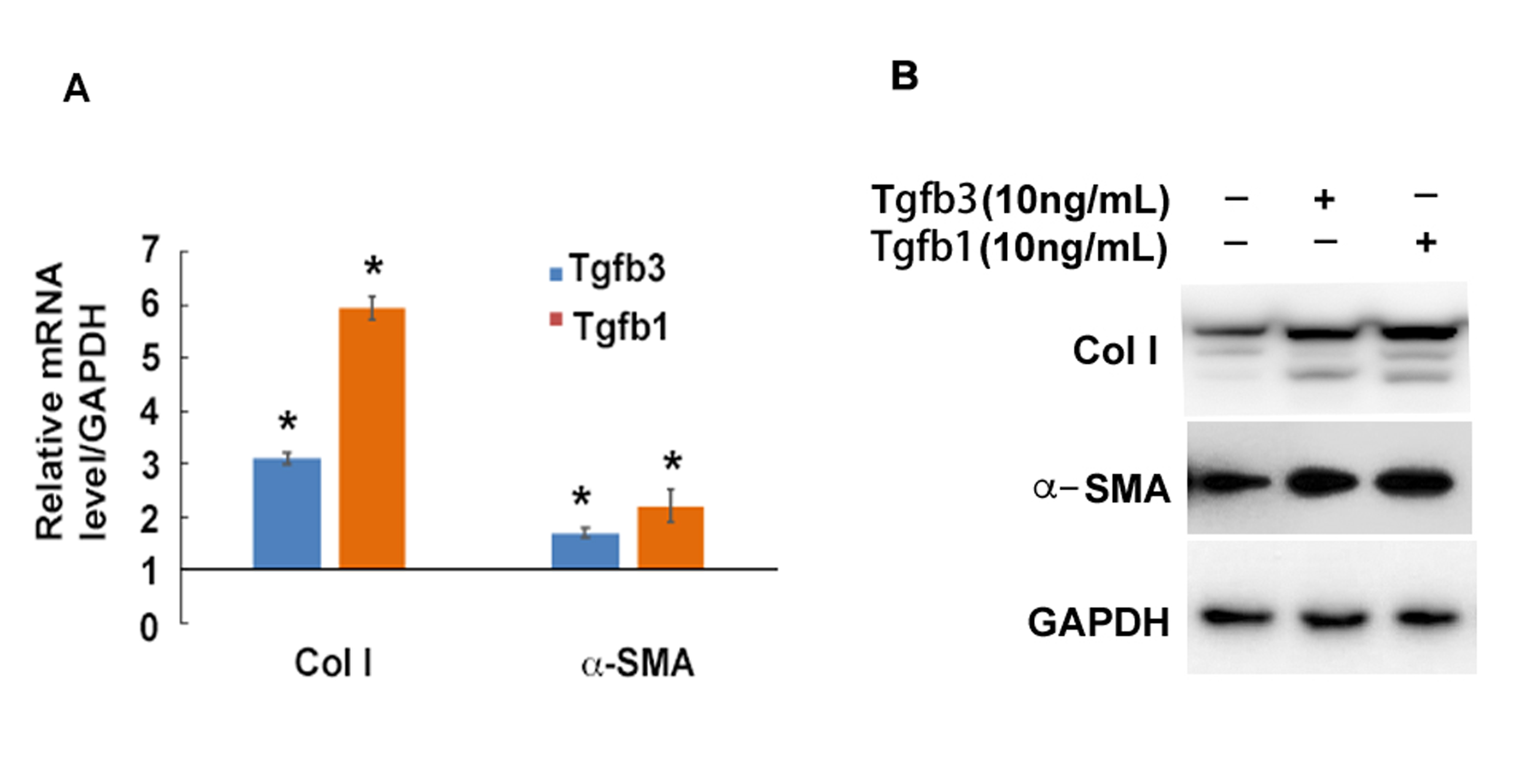

Supplement: Supplementary file 2 — Fig S2 [file JCMM-25-867-s002.jpg]

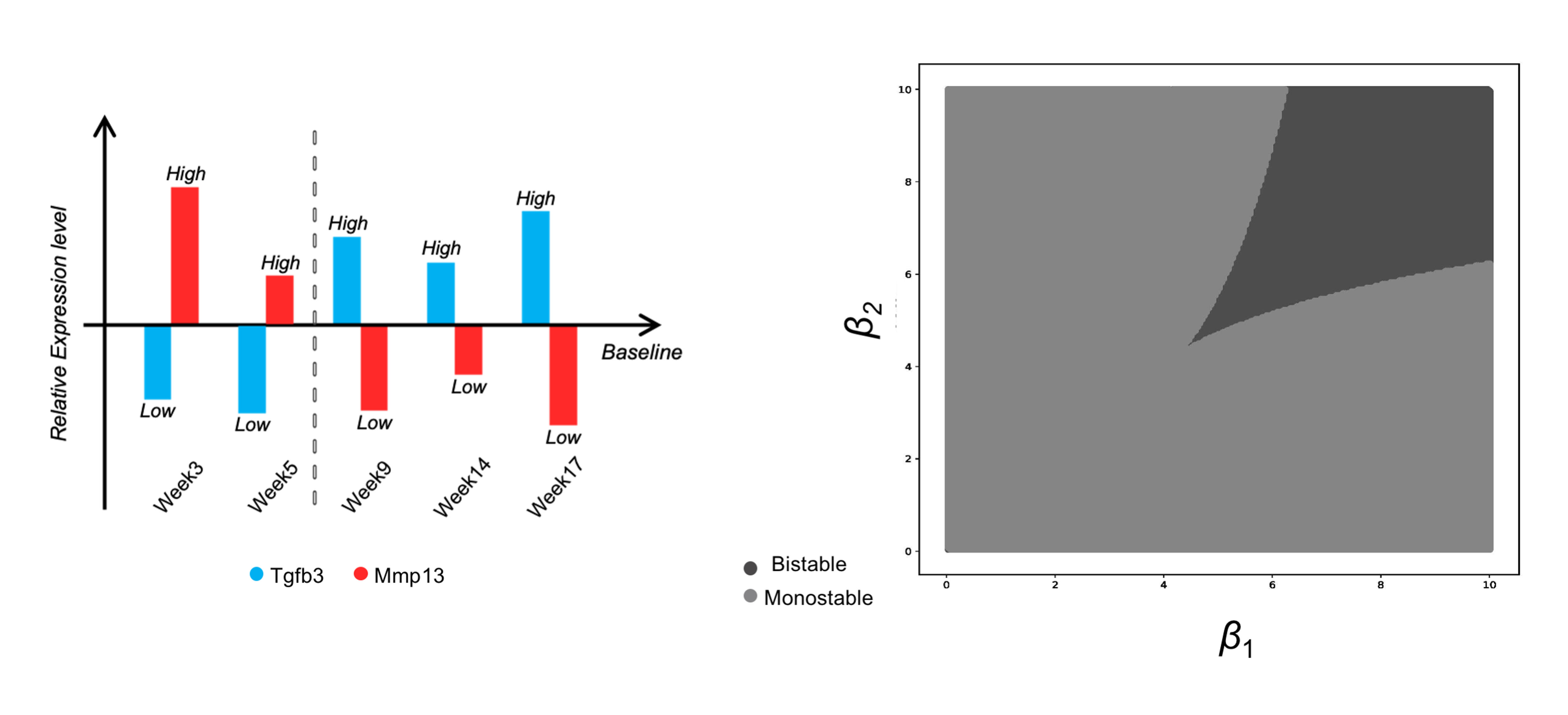

Supplement: Supplementary file 3 — Fig S3 [file JCMM-25-867-s003.jpg]
