# Supplementary material for: Tgfb3 and Mmp13 regulated the initiation of liver fibrosis progression as dynamic network biomarkers
Source: J Cell Mol Med. 2020 Dec 2;25(2):867–79. doi: 10.1111/jcmm.16140 (PMC7812286; doi:10.1111/jcmm.16140)
Supplement: Supplementary file 4 — Table S1‐S4 [file JCMM-25-867-s004.doc]

**Supplemental table 1. Fifty-two differentially expressed genes in the livers of mice before and after 9-week thioacetamide treatment present in dynamic network biomarkers (DNBs)**

| No. | ID | Symbol | Entrez gene name | Location | Type (s) |
| --- | --- | --- | --- | --- | --- |
| 1 | Akr1c6 | AKR1C4 | Aldo-keto reductase family 1 member C4 | Cytoplasm | Enzyme |
| 2 | Alpk1 | ALPK1 | Alpha kinase 1 | Other | Kinase |
| 3 | Arc | ARC | Activity-regulated cytoskeleton-associated protein | Cytoplasm | Other |
| 4 | C730036E19Rik | C730036E19Rik | RIKEN cdna C730036E19 gene | Other | Other |
| 5 | Car1 | CA1 | Carbonic anhydrase 1 | Cytoplasm | Enzyme |
| 6 | Ces1d | CES1 | Carboxylesterase 1 | Cytoplasm | Enzyme |
| 7 | Ces2a | Ces2a | Carboxylesterase 2A | Cytoplasm | Enzyme |
| 8 | Ces3b | CES3 | Carboxylesterase 3 | Cytoplasm | Enzyme |
| 9 | Ces3a | CES3 | Carboxylesterase 3 | Cytoplasm | Enzyme |
| 10 | Chil3 | Chil3/Chil4 | Chitinase-like 3 | Cytoplasm | Enzyme |
| 11 | Cxcl1 | CXCL2 | C-X-C motif chemokine ligand 2 | Extracellular space | Cytokine |
| 12 | Cyp1a1 | CYP1A1 | Cytochrome P450 family 1 subfamily A member 1 | Cytoplasm | Enzyme |
| 13 | Cyp2d13 | Cyp2d13 | Cytochrome P450, family 2, subfamily d, polypeptide 13 | Other | Other |
| 14 | Dio1 | DIO1 | Iodothyronine deiodinase 1 | Cytoplasm | Enzyme |
| 15 | Emilin2 | EMILIN2 | Elastin microfibril interfacer 2 | Extracellular space | Other |
| 16 | Fermt1 | FERMT1 | Fermitin family member 1 | Plasma membrane | Other |
| 17 | Fst | FST | Follistatin | Extracellular space | Other |
| 18 | Gprc5b | GPRC5B | G protein-coupled receptor class C group 5 member B | Plasma membrane | G-protein coupled receptor |
| 19 | Hacl1 | HACL1 | 2-hydroxyacyl-coa lyase 1 | Cytoplasm | Enzyme |
| 20 | Hsd17b6 | HSD17B6 | Hydroxysteroid 17-beta dehydrogenase 6 | Other | Enzyme |
| 21 | Id1 | ID1 | Inhibitor of DNA binding 1, HLH protein | Nucleus | Transcription regulator |
| 22 | Il1r2 | IL1R2 | Interleukin 1 receptor type 2 | Plasma membrane | Transmembrane receptor |
| 23 | Inmt | INMT | Indolethylamine N-methyltransferase | Cytoplasm | Enzyme |
| 24 | Irak1bp1 | IRAK1BP1 | Interleukin 1 receptor associated kinase 1 binding protein 1 | Other | Other |
| 25 | Itgb2l | Itgb2l | Integrin beta 2-like | Plasma membrane | Other |
| 26 | Kif1a | KIF1A | Kinesin family member 1A | Cytoplasm | Other |
| 27 | Klf5 | KLF5 | Kruppel like factor 5 | Nucleus | Transcription regulator |
| 28 | Lrp8 | LRP8 | LDL receptor related protein 8 | Plasma membrane | Transmembrane receptor |
| 29 | Ltbp2 | LTBP2 | Latent-transforming growth factor beta binding protein 2 | Extracellular space | Other |
| 30 | Ly6c2 | Ly6a (includes others) | Lymphocyte antigen 6 complex, locus A | Plasma membrane | Other |
| 31 | Megf6 | MEGF6 | Multiple EGF-like domain 6 | Cytoplasm | Other |
| 32 | Mex3a | MEX3A | Mex-3 RNA binding family member A | Other | Other |
| 33 | Mgat3 | MGAT3 | Mannosyl (beta-1,4-)-glycoprotein beta-1,4-N-acetylglucosaminyltransferase | Cytoplasm | Enzyme |
| 34 | Mmp13 | MMP13 | Matrix metallopeptidase 13 | Extracellular space | Peptidase |
| 35 | Mmp7 | MMP7 | Matrix metallopeptidase 7 | Extracellular space | Peptidase |
| 36 | Mrc2 | MRC2 | Mannose receptor C type 2 | Plasma membrane | Transmembrane receptor |
| 37 | Mtcl1 | MTCL1 | Microtubule crosslinking factor 1 | Cytoplasm | Other |
| 38 | Nfe2 | NFE2 | Nuclear factor, erythroid 2 | Nucleus | Transcription regulator |
| 39 | Nfkbiz | NFKBIZ | NFKB inhibitor zeta | Nucleus | Transcription regulator |
| 40 | Nudt7 | NUDT7 | Nudix hydrolase 7 | Cytoplasm | Enzyme |
| 41 | Orm3 | Orm1 (includes others) | Orosomucoid 1 | Extracellular space | Other |
| 42 | Pabpc1l | PABPC1L | Poly(A) binding protein cytoplasmic 1 like | Cytoplasm | Other |
| 43 | Pik3ip1 | PIK3IP1 | Phosphoinositide-3-kinase interacting protein 1 | Cytoplasm | Other |
| 44 | Rassf10 | RASSF10 | Ras association domain family member 10 | Other | Other |
| 45 | Retsat | RETSAT | Retinol saturase | Cytoplasm | Enzyme |
| 46 | Rnf138rt1 | Rnf138rt1 | Ring finger protein 138, retrogene 1 | Other | Other |
| 47 | Selenbp2 | SELENBP1 | Selenium binding protein 1 | Cytoplasm | Other |
| 48 | Sh3bp4 | SH3BP4 | SH3 domain binding protein 4 | Cytoplasm | Other |
| 49 | Sytl5 | SYTL5 | Synaptotagmin like 5 | Other | Other |
| 50 | Tgfb3 | TGFB3 | Transforming growth factor beta 3 | Extracellular space | Growth factor |
| 51 | Tmem86b | TMEM86B | Transmembrane protein 86B | Cytoplasm | Enzyme |
| 52 | Wfdc15b | Wfdc15b | WAP four-disulfide core domain 15B | Extracellular space | Other |

**Supplemental table 2. Existence of differentially expressed genes and dynamic network biomarkers (DNBs) in the potential fibrosis-associated pathways of the KEGG database**

| **Term** | **Count** | **X.** | **PValue** | **Genes** | **Fold Enrichment** | **Bonferroni** | **Benjamini** | **FDR** | **ID** |
| --- | --- | --- | --- | --- | --- | --- | --- | --- | --- |
| mmu00830 | 16 | 2.213001383 | 3.46E-07 | CYP3A16, CYP1A1, CYP1A2, CYP4A12B, UGT2B38, CYP4A10, RDH12, UGT1A9, CYP4A12A, CYP4A32, CYP3A41A, CYP4A31, AOX3, HSD17B6, RETSAT, CYP3A44 | 5.140241365 | 8.05E-05 | 4.03E-05 | 0.000444 | Retinol metabolism |
| mmu04512 | 12 | 1.659751037 | 0.000221 | COL4A4, COL4A3, LAMA5, ITGB8, LAMC3, ITGB6, ITGB4, ITGA11, LAMC2, THBS1, COL4A6, SPP1 | 3.898989899 | 0.050276374 | 0.008560524 | 0.284016442 | ECM-receptor interaction |
| mmu04510 | 19 | 2.627939142 | 0.000311 | COL4A4, PIK3CG, COL4A3, ITGA11, ITGB4, COL4A6, PAK6, PAK3, LAMC3, ITGB8, LAMA5, ITGB6, PDGFRA, LAMC2, PDGFD, THBS1, SHC2, SHC4, SPP1 | 2.624440866 | 0.069819489 | 0.010286247 | 0.398268322 | Focal adhesion |
| mmu04060 | 21 | 2.904564315 | 0.000336 | IL1R2, IL18RAP, OSMR, IL7, TNFRSF12A, CCR1, CXCL9, TGFB3, CXCR2, CX3CL1, TGFB2, CCR9, LIF, CXCL14, CXCL13, CLCF1, IL1RAP, CX3CR1, PDGFRA, PDGFD, IFNLR1 | 2.450793651 | 0.075420359 | 0.009754128 | 0.431430065 | Cytokine-cytokine receptor interaction |
| mmu03320 | 11 | 1.521438451 | 0.000426 | CYP4A10, CYP4A12B, LPL, CYP4A12A, SCD2, CYP4A32, CYP7A1, CYP4A31, FABP1, CYP8B1, ANGPTL4 | 3.931481481 | 0.094605481 | 0.010981975 | 0.546478454 | PPAR signaling pathway |
| mmu04390 | 13 | 1.798063624 | 0.006337027 | FZD9, GDF6, TGFB3, FZD3, TGFB2, WNT7B, ITGB2L, CTGF, ID1, WNT11, PPP2R2B, WNT7A, DLG2 | 2.461613932 | 0.772641102 | 0.107688882 | 7.842438411 | Hippo signaling pathway |
| mmu04350 | 9 | 1.244813278 | 0.00926817 | INHBB, INHBE, ID1, GDF6, FST, TGFB3, TGIF2, THBS1, TGFB2 | 3.02745098 | 0.885771348 | 0.119812892 | 11.27449765 | TGF-β signaling pathway |
| mmu04151 | 22 | 3.042876902 | 0.01064899 | COL4A4, PIK3CG, COL4A3, OSMR, IL7, ITGA11, ITGB4, FGF12, COL4A6, G6PC, LAMC3, ITGB8, LAMA5, ITGB6, PDGFRA, ANGPT1, LAMC2, EFNA4, PDGFD, THBS1, PPP2R2B, SPP1 | 1.792128311 | 0.917463973 | 0.129410287 | 12.85010093 | PI3K-Akt signaling pathway |
| mmu04062 | 14 | 1.93637621 | 0.019500171 | PIK3CG, CXCL1, ADCY1, ADCY8, CCR1, CXCL9, CXCR2, CX3CL1, CCR9, CXCL14, CXCL13, CX3CR1, SHC2, SHC4 | 2.042328042 | 0.98983113 | 0.196273617 | 22.35261494 | Chemokine signaling pathway |
| mmu04360 | 10 | 1.383125864 | 0.035878803 | SEMA5A, PAK6, UNC5B, PAK3, SEMA3C, SEMA3B, EFNA4, NFATC2, SLIT2, EPHB2 | 2.216480046 | 0.999799243 | 0.247068151 | 37.46257148 | Axon guidance |
| mmu04010 | 14 | 1.93637621 | 0.107663763 | IL1R2, NTF5, TGFB3, HSPA1A, HSPA1B, FGF12, TGFB2, CACNA2D4, DUSP5, MAPK13, NTRK2, PDGFRA, CACNA1H, CD14 | 1.5821988 | 1 | 0.405730717 | 76.85575607 | MAPK signaling pathway |
| mmu04723 | 7 | 0.968188105 | 0.149564488 | SLC17A8, ADCY1, ADCY8, MAPK13, MGLL, ITPR3, GABRP | 1.943185904 | 1 | 0.490366565 | 87.52308749 | Retrograde endocannabinoid signaling |
| mmu04974 | 6 | 0.829875519 | 0.192151655 | COL4A4, KCNN4, COL4A3, KCNQ1, COL4A6, KCNE3 | 1.949494949 | 1 | 0.529189378 | 93.55130219 | Protein digestion and absorption |
| mmu04310 | 8 | 1.106500692 | 0.220906344 | FZD9, WNT7B, NKD2, MMP7, FZD3, WNT11, NFATC2, WNT7A | 1.622274757 | 1 | 0.574856849 | 95.95182768 | Wnt signaling pathway |
| mmu04722 | 7 | 0.968188105 | 0.251954615 | NTRK3, PIK3CG, NTF5, MAPK13, NTRK2, SHC2, SHC4 | 1.640558591 | 1 | 0.594177466 | 97.59917817 | Neurotrophin signaling pathway |
| mmu04514 | 8 | 1.106500692 | 0.335147699 | NRCAM, CLDN8, CLDN7, ITGB2L, ITGB8, CLDN6, CDH3, ITGAM | 1.411979881 | 1 | 0.695430033 | 99.47208864 | Cell adhesion molecules |
| mmu04620 | 5 | 0.691562932 | 0.470203192 | PIK3CG, MAPK13, CXCL9, CD14, SPP1 | 1.415474881 | 1 | 0.80691447 | 99.97144716 | Toll-like receptor signaling pathway |
| mmu04630 | 6 | 0.829875519 | 0.573615429 | LIF, PIK3CG, IL7, OSMR, SOCS3, IFNLR1 | 1.183141762 | 1 | 0.851880262 | 99.99824579 | Jak-STAT signaling pathway |
| mmu01040 | 2 | 0.276625173 | 0.616812296 | SCD2, ACOT3 | 2.117969822 | 1 | 0.86648207 | 99.99955525 | Biosynthesis of unsaturated fatty acids |
| mmu04614 | 2 | 0.276625173 | 0.711801246 | ACE, CTSG | 1.633862434 | 1 | 0.897970338 | 99.99998855 | Renin-angiotensin system |
| mmu04621 | 2 | 0.276625173 | 0.863760298 | CARD9, MAPK13, CXCL1 | 1.021164021 | 1 | 0.959363567 | 100 | NOD-like receptor signaling pathway |
| mmu04066 | 3 | 0.414937759 | 0.882770159 | PIK3CG, HKDC1, ANGPT1 | 0.824786325 | 1 | 0.961782634 | 100 | HIF-1 signaling pathway |
| mmu04920 | 2 | 0.276625173 | 0.923126102 | G6PC, SOCS3 | 0.794238683 | 1 | 0.97445658 | 100 | Adipocytokine signaling pathway |
| mmu04064 | 1 | 0.138312586 | 1 | CD14 | 0.294768996 | 1 | 1 | 100 | NF-kappa B signaling pathway |
| mmu04340 | 1 | 0.138312586 | 1 | IHH | 1.191358025 | 1 | 1 | 100 | Hedgehog signaling pathway |

**Supplemental table 3. Existence of differentially expressed genes and dynamic network biomarkers (DNBs) in the potential fibrosis-associated pathways of IPA database**

| **Ingenuity Canonical Pathways** | **-log(p-value)** | **Ratio** | **z-score** | **Molecules** |
| --- | --- | --- | --- | --- |
| Retinol Biosynthesis | 2.87 | 0.167 | #NUM! | AADAC, LIPC, CES1D, LPL, RDH12, LIPG |
| Retinol Biosynthesis | 2.64 | 0.0833 | #NUM! | Ces1e, PNPLA5, CES1D |
| Retinol Biosynthesis | 1.08 | 0.0278 | #NUM! | CES1D |
| Granulocyte Adhesion and Diapedesis | 6.48 | 0.131 | #NUM! | MMP7, MMP13, CLDN7, MMP25, CLDN6, Cxcl9, IL18RAP, IL1R2, IL36G, ITGAM, CLDN8, CXCL13, CXCR2, MMP8, EZR, CXCL14, CXCL1, IL1RAP, CX3CL1, MMP9 |
| Granulocyte Adhesion and Diapedesis | 3.04 | 0.0392 | #NUM! | IL1R2, MMP7, MMP13, MMP12, CXCL1, HSPB1 |
| Granulocyte Adhesion and Diapedesis | 3.31 | 0.0261 | #NUM! | IL1R2, MMP7, MMP13, CXCL1 |
| Role of JAK family kinases in IL-6-type Cytokine Signaling | 1.27 | 0.12 | #NUM! | SOCS3, OSMR, MAPK13 |
| Agranulocyte Adhesion and Diapedesis | 3.76 | 0.0982 | #NUM! | MMP7, MMP13, CLDN7, MMP25, CLDN6, Cxcl9, IL36G, CXCL13, CLDN8, CXCR2, EZR, MMP8, CXCL14, CXCL1, CX3CL1, MMP9 |
| Agranulocyte Adhesion and Diapedesis | 2.16 | 0.0307 | #NUM! | MMP7, ACTA2, MMP13, MMP12, CXCL1 |
| Agranulocyte Adhesion and Diapedesis | 2.16 | 0.0184 | #NUM! | MMP7, MMP13, CXCL1 |
| STAT3 Pathway | 1.89 | 0.0959 | 1.89 | MAP3K9, SOCS3, NTRK2, NTRK3, PDGFRA, MAPK13, DDR1 |
| STAT3 Pathway | 0.382 | 0.0137 | #NUM! | PDGFRB |
| Hepatic Fibrosis / Hepatic Stellate Cell Activation | 3.38 | 0.0909 | #NUM! | CTGF, COL4A6, COL4A3, KLF6, MMP13, COL28A1, IL18RAP, IL1R2, TGFB2, TGFB3, PDGFRA, CD14, COL4A4, PDGFD, IL1RAP, MMP9 |
| Hepatic Fibrosis / Hepatic Stellate Cell Activation | 4.34 | 0.0455 | #NUM! | IL1R2, COL1A1, COL6A2, ACTA2, TGFB3, MMP13, PDGFRB, COL3A1 |
| Hepatic Fibrosis / Hepatic Stellate Cell Activation | 2.07 | 0.017 | #NUM! | IL1R2, TGFB3, MMP13 |
| Wnt/Ca+ pathway | 1.33 | 0.0877 | 2 | PLCD3, FZD3, NFATC2, FZD9, ROR1 |
| HIF1α Signaling | 1.81 | 0.0804 | #NUM! | SLC2A5, MMP7, PIK3CG, MMP8, MMP13, MAPK13, MMP25, MMP9, SLC2A3 |
| HIF1α Signaling | 1.31 | 0.0268 | #NUM! | MMP7, MMP13, MMP12 |
| HIF1α Signaling | 1.53 | 0.0179 | #NUM! | MMP7, MMP13 |
| Wnt/β-catenin Signaling | 2.31 | 0.0788 | 0.302 | SOX4, MMP7, WNT7A, FRZB, CDH3, DKK3, FZD3, WNT7B, PPP2R2B, TGFB3, TGFB2, FZD9, WNT11 |
| Wnt/β-catenin Signaling | 0.469 | 0.0121 | #NUM! | MMP7, TGFB3 |
| Wnt/β-catenin Signaling | 1.23 | 0.0121 | #NUM! | MMP7, TGFB3 |
| Renin-Angiotensin Signaling | 1.67 | 0.0763 | 3 | PAK6, PAK3, PIK3CG, ADCY1, SHC2, ITPR3, MAPK13, ADCY8, ACE |
| Role of hypercytokinemia/hyperchemokinemia in the Pathogenesis of Influenza | 0.598 | 0.0714 | #NUM! | CCR1, IL36G |
| Axonal Guidance Signaling | 3.47 | 0.0682 | #NUM! | MMP7, NTF4, FZD3, EPHB2, UNC5B, MMP13, TUBB2B, PLCD3, WNT7A, MMP8, WNT7B, PIK3CG, SEMA3B, ACE, ADAMTS4, PAK6, SEMA5A, FZD9, GNAZ, SLIT2, EFNA4, NTRK2, PAK3, GLIS2, NTRK3, NFATC2, SEMA3C, PDGFD, MMP9, WNT11 |
| Axonal Guidance Signaling | 0.546 | 0.00455 | #NUM! | MMP7, MMP13 |
| Neurotrophin/TRK Signaling | 0.929 | 0.0667 | #NUM! | NTRK2, NTF4, NTRK3, PIK3CG, SPRY2 |
| PPAR Signaling | 1.02 | 0.0659 | -1.633 | IL1R2, IL36G, PDGFRA, PDGFD, IL1RAP, IL18RAP |
| PPAR Signaling | 0.846 | 0.022 | #NUM! | IL1R2, PDGFRB |
| PPAR Signaling | 0.707 | 0.011 | #NUM! | IL1R2 |
| p38 MAPK Signaling | 1.05 | 0.0631 | 1.89 | IL1R2, IL36G, TGFB3, TGFB2, MAPK13, IL1RAP, IL18RAP |
| p38 MAPK Signaling | 1.32 | 0.027 | #NUM! | IL1R2, TGFB3, HSPB1 |
| p38 MAPK Signaling | 1.54 | 0.018 | #NUM! | IL1R2, TGFB3 |
| Differential Regulation of Cytokine Production in Intestinal Epithelial Cells by IL-17A and IL-17F | 0.347 | 0.0588 | #NUM! | LCN2 |
| PPARα/RXRα Activation | 0.879 | 0.0533 | -0.816 | IL1R2, PLCD3, LPL, ADCY1, TGFB3, TGFB2, ADCY8, IL1RAP, IL18RAP |
| PPARα/RXRα Activation | 0.456 | 0.0118 | #NUM! | IL1R2, TGFB3 |
| PPARα/RXRα Activation | 1.21 | 0.0118 | #NUM! | IL1R2, TGFB3 |
| UVC-Induced MAPK Signaling | 0.368 | 0.0476 | #NUM! | MAPK13, SMPD3 |
| TGF-β Signaling | 0.47 | 0.0471 | 2 | TGFB3, TGFB2, MAPK13, INHBB |
| TGF-β Signaling | 0.893 | 0.0235 | #NUM! | TGFB3, INHBA |
| TGF-β Signaling | 0.733 | 0.0118 | #NUM! | TGFB3 |
| Signaling by Rho Family GTPases | 0.677 | 0.0458 | 2.53 | MAP3K9, NOX4, PAK6, PAK3, CDH3, CDC42EP5, EZR, PIK3CG, CDH6, ARHGEF17, GNAZ |
| Role of JAK1 and JAK3 in γc Cytokine Signaling | 0.359 | 0.0429 | #NUM! | SOCS3, PIK3CG, IL7 |
| Toll-like Receptor Signaling | 0.359 | 0.0429 | #NUM! | IL36G, CD14, MAPK13 |
| NF-κB Signaling | 0.415 | 0.0407 | 2.646 | IL1R2, IL36G, NTRK2, NTRK3, PIK3CG, PDGFRA, DDR1 |
| NF-κB Signaling | 0.446 | 0.0116 | #NUM! | IL1R2, PDGFRB |
| NF-κB Signaling | 0.469 | 0.00581 | #NUM! | IL1R2 |
| UVA-Induced MAPK Signaling | 0.334 | 0.0396 | #NUM! | PLCD3, PIK3CG, MAPK13, SMPD3 |
| Integrin Signaling | 0.351 | 0.0379 | 2.828 | ITGAM, PAK6, PAK3, ITGA11, PIK3CG, ITGB4, ITGB8, ITGB6 |
| Integrin Signaling | 0.34 | 0.00948 | #NUM! | ACTA2, ITGA7 |
| LPS-stimulated MAPK Signaling | 0.253 | 0.0357 | #NUM! | PIK3CG, CD14, MAPK13 |
| HIPPO Signaling | 0.253 | 0.0357 | #NUM! | PPP2R2B, DLG2, SFN |
| Sonic Hedgehog Signaling | 0.203 | 0.0357 | #NUM! | GLIS2 |
| RhoA Signaling | 0.239 | 0.00855 | #NUM! | ACTA2 |
| Regulation of Actin-based Motility by Rho | 0.337 | 0.0119 | #NUM! | ACTA2 |
| Fatty Acid α-oxidation | 0.866 | 0.05 | #NUM! | ALDH1A2 |

**Supplemental table 4. Gene Ontology (GO) annotations related to the 12 dynamic network biomarker (DNB) genes and related pathways**

| **Symbol** | **Entrez Gene Name** | **GO annotations related to this gene** | **Related pathways** |
| --- | --- | --- | --- |
| IL1R2 | Interleukin 1 receptor type 2 | A non-signaling receptor for IL1A, IL1B, and IL1RN, reduces IL1B activities, serves as a decoy receptor by competitive binding to IL1B and preventing its binding to IL1R1 | Human T-cell leukemia virus 1 infection and innate immune system |
| FST | Follistatin | Obsolete signal transducer activity and activin binding | TGF-β signaling pathway and TGF-b receptor signaling |
| HSD17B6 | Hydroxysteroid 17-Beta Dehydrogenase 6 | Oxidoreductase activity and retinol dehydrogenase activity. Has retinol dehydrogenase activity towards all-trans-retinol (in vitro) | Drug metabolism - cytochrome P450 and steroid hormone biosynthesis |
| MMP7 | Matrix metallopeptidase 7 | Encodes a member of the peptidase M10 family of matrix metalloproteinases (MMPs). Cell adhesion_ECM remodeling and collagen chain trimerization | Peptidase activity and metallopeptidase activity |
| MMP13 | Matrix metalloproteinase 13 | Zinc-dependent endopeptidases and major proteases in extracellular matrix proteins, ECM degradation; plays a role in the degradation of including fibrillar collagen, fibronectin, TNC, and ACAN; may also function by activating or degrading key regulatory proteins, such as TGFB1 and CTGF | Cell adhesion, ECM remodeling and collagen chain trimerization. |
| Id1 | Inhibitor of DNA binding 1, HLH protein | A helix-loop-helix (HLH) protein that can form heterodimers with members of the basic HLH family of transcription factors. The encoded protein has no DNA binding activity and therefore can inhibit the DNA binding and transcriptional activation ability of basic HLH proteins with which it interacts. This protein may play a role in cell growth, senescence, and differentiation apoptosis, angiogenesis, and neoplastic transformation | ID signaling pathway and Hippo signaling pathway |
| Cyp1a1 | Cytochrome P450 family 1 subfamily A member 1 | Encodes a member of the cytochrome P450 superfamily of enzymes. The cytochrome P450 proteins are monooxygenases which catalyze many reactions involved in drug metabolism and synthesis of cholesterol, steroids, and other lipids. It oxidizes various structurally unrelated compounds, including steroids, fatty acids, and xenobiotics | Drug metabolism - cytochrome P450 and Cytochrome P450 - arranged by substrate type |
| Cxcl1 | C-X-C motif chemokine ligand 1 | Signaling receptor binding and chemokine activity | Interleukin-10 signaling and TNF signaling pathway. |
| Retsat | Retinol saturase | Oxidoreductase activity and all-trans-retinol 13,14-reductase activity. May play a role in the metabolism of vitamin A. Independently of retinol conversion, it may regulate liver metabolism upstream of MLXIPL/ChREBP. May play a role in adipocyte differentiation | Drug metabolism - cytochrome P450 and Vitamin A and Carotenoid Metabolism |
| Itgb2L | Integrin subunit beta 2 | Encodes an integrin beta chain, which combines with multiple different alpha chains to form different integrin heterodimers. Integrins are integral cell-surface proteins that participate in cell adhesion as well as cell-surface mediated signaling | Cytokine signaling immune system and complement and coagulation cascades |
| Cxcl2(Cxcl1) | C-X-C motif chemokine ligand 1 | This antimicrobial gene encodes a member of the CXC subfamily of chemokines with receptor binding and chemokine activity. The encoded protein is a secreted growth factor that signals through the G-protein coupled receptor, CXC receptor 2. This protein plays a role in inflammation and as a chemoattractant for neutrophils | PEDF induced signaling and cytokine signaling in immune system |
| Ces1 | Ces1d | Encodes a member of the carboxylesterase large family with hydrolase activity and methylumbelliferyl-acetate deacetylase activity. The family members are responsible for the hydrolysis or transesterification of various xenobiotics, and endogenous substrates with ester, thioester, or amide bonds. This enzyme is the major liver enzyme and functions in liver drug clearance | Phase I biotransformations, non P450 and Cholesterol and Sphingolipids transport / Transport from Golgi and ER to the apical membrane (normal and CF) |
